# *N*-Alkylaminoferrocene-Based Prodrugs Targeting Mitochondria of Cancer Cells

**DOI:** 10.3390/molecules25112545

**Published:** 2020-05-29

**Authors:** Viktor Reshetnikov, Hülya Gizem Özkan, Steffen Daum, Christina Janko, Christoph Alexiou, Caroline Sauer, Markus R. Heinrich, Andriy Mokhir

**Affiliations:** 1 Organic Chemistry Chair II, Department of Chemistry and Pharmacy, Friedrich-Alexander University Erlangen-Nürnberg (FAU), Nikolaus-Fiebiger-Str. 10, 91058 Erlangen, Germany; reshviktor1@gmail.com (V.R.); huelya.gizem.oezkan@fau.de (H.G.Ö.); Steffen.Daum@gmail.com (S.D.); 2 Merck, Im Laternenacker 5, 8200 Schaffhausen, Switzerland; 3 Department of Otorhinolaryngology, Head and Neck Surgery, Section of Experimental Oncology and Nanomedicine (SEON), Else Kröner-Fresenius-Stiftung-Professorship, Universitätsklinikum Erlangen, Glückstraße 10a, 91054 Erlangen, Germany; christina.janko@uk-erlangen.de (C.J.); christoph.alexiou@uk-erlangen.de (C.A.); 4 Medicinal Chemistry, Department of Chemistry and Pharmacy, Friedrich-Alexander University Erlangen-Nürnberg (FAU), Nikolaus-Fiebiger-Str. 10, 91058 Erlangen, Germany; caroline.sauer@fau.de (C.S.); markus.heinrich@fau.de (M.R.H.)

**Keywords:** *N*-alkylaminoferrocene, prodrug, cancer, mitochondrion, reactive oxygen species

## Abstract

Intracellular concentration of reactive oxygen species (e.g., H_2_O_2_) in cancer cells is elevated over 10-fold as compared to normal cells. This feature has been used by us and several other research groups to design cancer specific prodrugs, for example, *N*-alkylaminoferrocene (NAAF)-based prodrugs. Further improvement of the efficacy of these prodrugs can be achieved by their targeting to intracellular organelles containing elevated reactive oxygen species (ROS) amounts. For example, we have previously demonstrated that lysosome-targeted NAAF-prodrugs exhibit higher anticancer activity in cell cultures, in primary cells and in vivo (Angew. Chem. Int. Ed. 2017, 56, 15545). Mitochondrion is an organelle, where electrons can leak from the respiratory chain. These electrons can combine with O_2_, generating O_2_^−•^ that is followed by dismutation with the formation of H_2_O_2_. Thus, ROS can be generated in excess in mitochondria and targeting of ROS-sensitive prodrugs to these organelles could be a sensible possibility for enhancing their efficacy. We have previously reported on NAAF-prodrugs, which after their activation in cells, are accumulated in mitochondria (Angew. Chem. Int. Ed. 2018, 57, 11943). Now we prepared two hybrid NAAF-prodrugs directly accumulated in mitochondria and activated in these organelles. We studied their anticancer activity and mode of action. Based on these data, we concluded that ROS produced by mitochondria is not available in sufficient quantities for activation of the ROS-responsive prodrugs. The reason for this can be efficient scavenging of ROS by antioxidants. Our data are important for the understanding of the mechanism of action of ROS-activatable prodrugs and will facilitate their further development.

## 1. Introduction

**Scheme 1:**
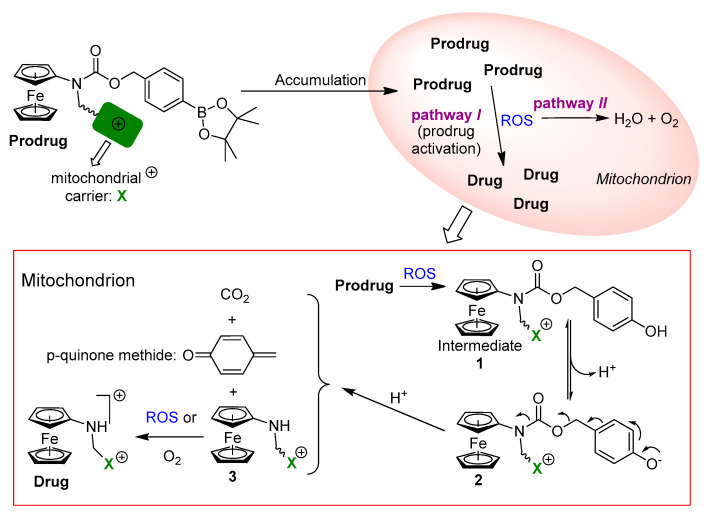
*N*-alkylaminoferrocene-(NAAF) based anticancer prodrugs targeting mitochondria due to a covalently attached carrier moiety X^+^. Intermediates **1**, **2**, **3**, p-quinone methide and CO_2_ as well as the drug are formed from the prodrug in the medium containing high amounts of reactive oxygen species (reactive oxygen species (ROS), e.g., in cancer cells).

Chemotherapy along with surgery and radiotherapy remains the most efficient and broadly applied method of cancer treatment [1]. However, despite the fact that some types of cancers are well responsive (e.g., testicular cancer to Pt(II)-based drugs [2]), chemotherapy still suffers from massive side effects and development of resistance in many other cases [3]. Therefore, the search for new chemotherapeutic approaches lacking the mentioned drawbacks is warranted. One sensible possibility is to use prodrugs, which are activated under cancer specific conditions, but remain inactive and therefore nontoxic in normal cells. For example, the intracellular concentration of reactive oxygen species (ROS) (e.g., hydrogen peroxide, H_2_O_2_) in most cancer cells is elevated by more than 10-fold in comparison to normal cells [4,5,6]. This difference in the chemical composition of cellular media was already used by us [7,8,9,10,11] and other research groups [12,13,14,15,16,17] to design experimental cancer-specific prodrugs activated by H_2_O_2_. In particular, we developed *N*-alkylaminoferrocene-(NAAF) based prodrugs, which are activated in the presence of ROS with the formation of NAAF drugs (Scheme 1). The latter drugs amplify the ROS concentration in cancer cells, leading to their death via apoptotic and necrotic pathways. We demonstrated that NAAF-prodrugs exhibit anticancer activity towards a range of cancer cell lines [7,8,9,10,11], in vivo in several mice [9,10,18] and rat cancer models [11] as well as towards primary cancer cells [8,10,18]. 

Since ROS are distributed heterogeneously in cells, targeting oxidatively activated prodrugs to intracellular areas with high amounts of ROS can be a feasible strategy for the enhancement of their efficacy. We have recently demonstrated the validity of this approach for the NAAF-prodrugs by re-directing them to lysosomes (LY’s) [18], which are known to accumulate ROS as well as redox active transition metal ions. We have found that the LY-targeting NAAF-prodrugs are activated much more efficiently in cancer cells than their non-targeted analogs, which is reflected in their higher anticancer activity in cell cultures, primary cells and in vivo [18]. Apart from LY’s, mitochondria of cancer cells are also considered as an important source of intracellular ROS due to leakage of electrons in the respiratory chain. However, in contrast to LY’s lacking catalase and peroxidases, mitochondria are well protected by the ROS-neutralizing enzymes. Therefore, it is not clear whether mitochondria-derived ROS are available in cells in sufficient amounts to be explored for prodrug activation (pathway I in Scheme 1) or they are rather neutralized by the cellular antioxidative system shortly after their formation (pathway II, Scheme 1). We have previously reported on NAAF-prodrugs, which are activated by ROS in the cytoplasm of cancer cells with the generation of lipophylic cationic drugs. The latter drugs are accumulated in mitochondria [19]. In contrast, herein we report on prodrugs, which are first accumulated in mitochondria and can then react with mitochondrial ROS (Scheme 1). Using these prodrugs, we clarified an important question of whether mitochondria-targeting of oxidatively activated NAAF-prodrugs favorably affects their anticancer activity. The results obtained are relevant to all other types of ROS-responsive prodrugs. They will facilitate the further development of these compounds as cancer specific drugs. 

## 2. Results and Discussion

### 2.1. Design, Synthesis and Basic Properties of Prodrugs in Cell Free Settings

**Scheme 2:**
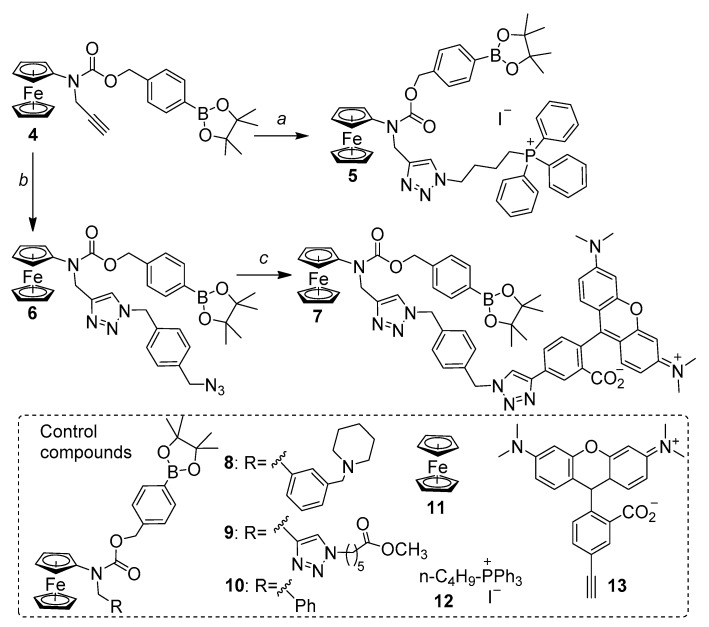
Synthesis of mitochondria-targeting prodrugs **5** and **7** as well as structures of the corresponding controls **8**–**13**: (a) (4-azidobutyl)triphenylphosphonium iodide [20], CuI, DMSO, 60 °C, 3 h; (b) 1,4-bis(azidomethyl)benzene, Cu(PPh_3_)_3_Br, CH_2_Cl_2_, 25 °C, 24 h; (c) compound **13**, CuI, 55 °C, 4 h followed by 25 °C, 16 h.

We conjugated a representative NAAF prodrug **4** to two known mitochondria-carriers (alkyltriphenylphosphonium (Ph_3_P-R^+^) [20] and *N*,*N*,*N*′,*N*′-tetramethylrhodamine (TMR^+^)) by using Cu^+^-catalyzed azide-alkyne cycloaddition (“click” reaction) leading to prodrugs **5** and **7** (Scheme 2). Additionally, control compounds (lysosome-targeting prodrug **8** [18], non-targeted prodrugs **9** [21] and **10** [7,8] as well as “empty” carriers **12** [22] and **13**) were prepared as described in the supporting information (SI). Our carrier selection was based on the following considerations. Both chosen carriers belong to the compound class called delocalized lipophilic cations (DLC’s). DLC’s are efficiently accumulated in mitochondria of cancer cells, in which the membrane potential is usually strongly negatively charged [22]. The carrier Ph_3_P-R^+^ has been broadly used in the past to design mitochondria-targeting drugs. However, its important drawback is rather high unspecific toxicity (cytotoxicity data for control **12**, Table 1). In contrast, TMR^+^ derivatives are less toxic (cytotoxicity data for control **13**, Table 1) and fluorescent, which allows convenient monitoring of their uptake by cells. In the conjugate of TMR with an *N*-alkylaminoferrocene fragment, we expected to observe at least partial quenching of TMR fluorescence due to photo-induced electron transfer (PET) from the ferrocene moiety. Upon activation of this compound in cancer cells with formation of ferrocenium species, the quenching would be released leading to fluorescent TMR. This would offer an additional possibility for investigation of the mechanism of prodrug activation both in cell free medium and in cells by monitoring fluorescence. 

**Figure 1:**
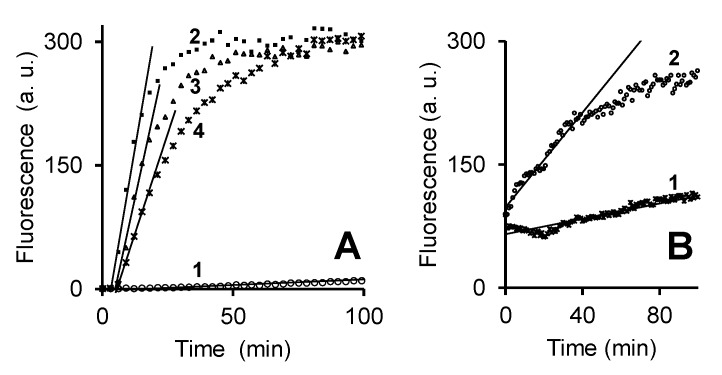
(**A**) Increase of fluorescence (λ_ex_ = 501 nm, λ_em_ = 525 nm) of solutions containing a mixture of 2′,7′-dichlorofluorescin (10 μM), 3-(N-morpholino)propanesulfonic acid (MOPS, 100 mM, pH 7.5), *N*,*N*,*N*′,*N*′-ethylenediaminetetracetic acid (EDTA, 10 mM), glutathione (GSH, 5 mM), H_2_O_2_ (10 mM, curve 1, negative control) and either FeSO_4_ (49.5 μM, curve 2, positive control) or prodrug **8** (49.5 μM, curve 3) or prodrug **5** (49.5 μM, curve 4); (dF/dt)_0_ = 22.3, 17.1, 7.7 and 0.1 a.u. × min^−1^; temperature = 37 °C. (**B**) Increase of fluorescence (λ_ex_ = 551 nm, λ_em_ = 576 nm) of solutions containing a mixture of prodrug **7** (0.5 μM), phosphate-buffered saline (10 mM phosphate, 150 mM NaCl, pH 7) and either without (curve 1, negative control) or with H_2_O_2_ (50 mM, curve 2). (dF/dt)_0_ = 0.5 (curve 1), 2.9 (curve 2) a.u. × min^−1^.

First, we evaluated the solubility of new prodrugs in representative aqueous buffers. In particular, we found that both compounds are moderately soluble reaching 70 + 15 (**5**), 44 + 4 (**7**) μM in Dulbecco’s phosphate-buffered saline (DPBS) containing DMSO (1%, *v*/*v*) and 130 + 17 (**5**), 47 + 4 (**7**) μM in Roswell Park Memorial Institute (RPMI) medium containing 20% of fetal bovine serum (FBS) and DMSO (1%, *v*/*v*) (Table S1, SI). Next, we confirmed that prodrug **5** is an efficient catalyst in the reaction of generation of highly toxic HO• from less toxic H_2_O_2_ (Figure 1A). In the latter assay, we applied 2′,7′-dichlorofluorescin as a probe (Table S2, SI). Prodrug **5** accelerates the initial rate ((dF/dt)_0_ (where F = fluorescence at λ = 531 nm expressed in arbitrary units (a.u.), λ_ex_ = 501 nm, t = time, min) of 2′,7′-dichlorofluorescin oxidation in the presence of H_2_O_2_ (*p* < 0.01, *n* = 3) from 0.1 a.u. × min^−1^ (curve 1, no drug added) to (dF/dt)_0_ = 7.7 a.u. × min^−1^ (curve 4, [5] = 49.5 μM). Known LY-targeting prodrug **8** [18] (curve 3, Figure 1A) and positive control FeSO_4_ (curve 2, Figure 1A) were found to be only slightly more active in this assay (in both cases *p* < 0.05, *n* = 3): 2.2- and 2.9-fold correspondingly. The reactivity of **5** is comparable to that of previously reported non-targeted prodrug **9** [21] and substantially higher than that of prodrug **10**, which is known to be prone to aggregation [7,8].

**Figure 2:**
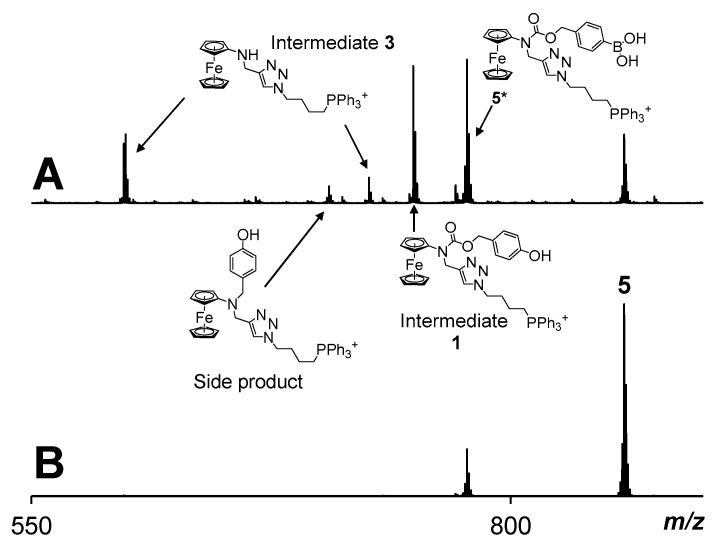
Mass spectra (electrospray ionization (ESI), positive mode) of prodrug **5** (40 μM in *N*,*N*-dimethylformamide DMF/CH_3_CN/H_2_O, 1/10/90, *v*/*v*/*v*) kept at 22 °C for 5 min in presence of H_2_O_2_ (35 mM, inset **A**) and in its absence (inset **B**). More detailed information (e.g., zoomed in spectra) is provided in the SI (Figure S15).

To determine active species responsible for the ROS generation, we acquired electrospray ionization (ESI) mass spectra of prodrug **5** with and without H_2_O_2_ (Figure 2, Figure S15). 

We could detect prominent peaks corresponding to hydrolyzed prodrug **5** (5*) as well intermediates **1** and **3** (two peaks corresponding to [M]^+^ and [M-e^−^ + I^−^]^+^ were observed for **3**) in the mass spectrum of the mixture of **5** and H_2_O_2_, whereas in the absence of H_2_O_2_ only intact (**5**) and hydrolyzed (5*) species were observed. These data confirm the mechanism of prodrug **5** activation outlined in Scheme 1. The ROS-generation assay (Figure 1A) could not be accurately conducted for prodrug **7** due to its own, TMR^+^-derived emission in the spectral range where 2′,7′-dichlorofluorescein (the product of 2′,7′-dichlorofluorescin oxidation) is detected. Fortunately, prodrug **7** activation can be directly monitored without any probe: the initial rate of fluorescence increase ((dF/dt)_0_, F: λ_ex_ = 551 nm, λ_em_ = 576 nm) of solution of **7** is 6-fold higher in the presence of H_2_O_2_ than in its absence (Figure 1B). The reason for that is that in the initial state (intact **7**) the fluorescence of the TMR^+^ dye is partially quenched due to the photo-induced electron transfer (PET) from the ferrocene moiety, whereas the quenching is released in the activated probe containing a ferrocenium moiety (drug in Scheme 1). Possible equilibrium between open (fluorescent) and closed (nonfluorescent) forms of TMR should be also considered. Since it is pH dependent and all our experiments are conducted in buffered aqueous solutions at the constant pH, contribution of this equilibrium to the final fluorescence increase will be canceled out. Thus, the described above data obtained in cell free settings indicate that conjugation of positively charged moieties to the NAAF-prodrug moiety does not substantially change its ability to react with H_2_O_2_ with the formation of drugs able to catalyze ROS amplification.

### 2.2. Study of Cellular Effects of Prodrugs and Control Compounds

**Figure 3:**
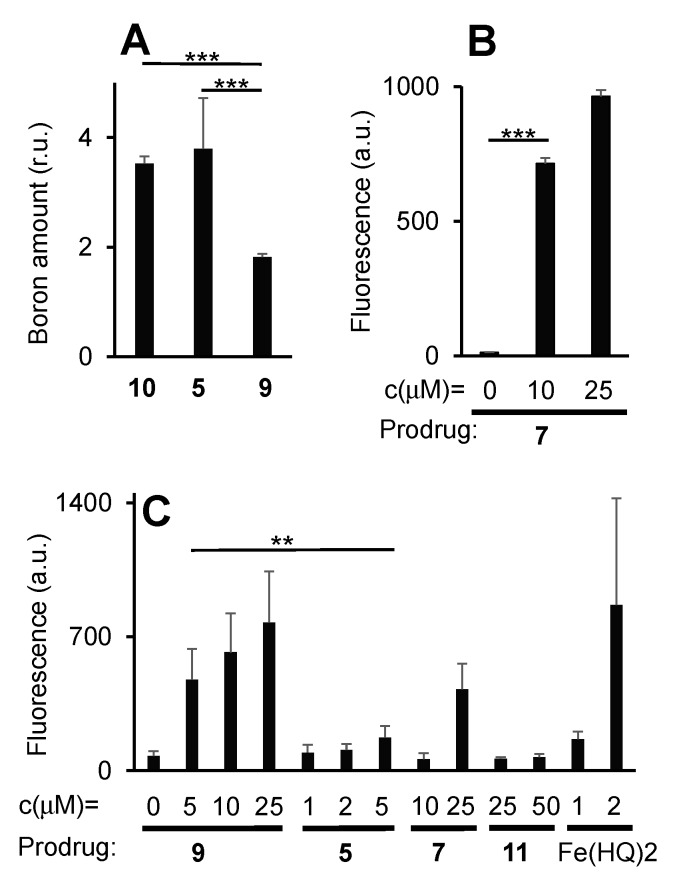
(**A**) Accumulation of prodrugs **5**, **9** and **10** (each 1 μM, incubation time- 1 h, temperature- 37 °C) in Burkitt’s lymphoma BL-2 cells monitored by analysis of the intracellular concentration of boron (Table S3, SI). (**B**) Uptake of fluorescent prodrug **7** (0–25 μM, incubation time—2 h, temperature—37 °C) by BL-2 cells monitored by flow cytometry (λ_ex_ = 488 nm, λ_em_= 530 nm). (**C**) Monitoring ROS-release in BL-2 cells (using 5(6-)chloromethyl-2′,7′-dichlorodihydrofluorescein diacetate as a fluorogenic probe, 10 μM) in the presence of prodrugs **5**, **7**, **9** as well as controls **11** (negative control) and a mixture of FeCl_3_ with 8-hydroxyquinoline (HQ) with a molar ratio of components correspondingly 1 to 2 (Fe(HQ)_2_, positive control); incubation time—2 h, temperature—37 °C. Student’s *t*-test: **—*p* < 0.01; ***—*p* < 0.001.

To test the cellular effects of the new prodrugs, we first studied whether these compounds are uptaken by cells. For these experiments, we selected a representative cancer cell line Burkitt’s lymphoma BL-2. Since natural boron concentration in these cells is negligible and prodrug **5** contains one boron atom per molecule, we could study the uptake of **5** by monitoring the amount of intracellular boron using the photometric curcumin-based assay as described in the *SI*. We observed that the efficiency of the uptake of **5** is 2-fold higher than that of known non-targeted prodrug **9** (*p* < 0.001, *n* = 3) and is comparable to that of LY-targeting prodrug **8** (Figure 3A, Table S3, SI). 

**Figure 4:**
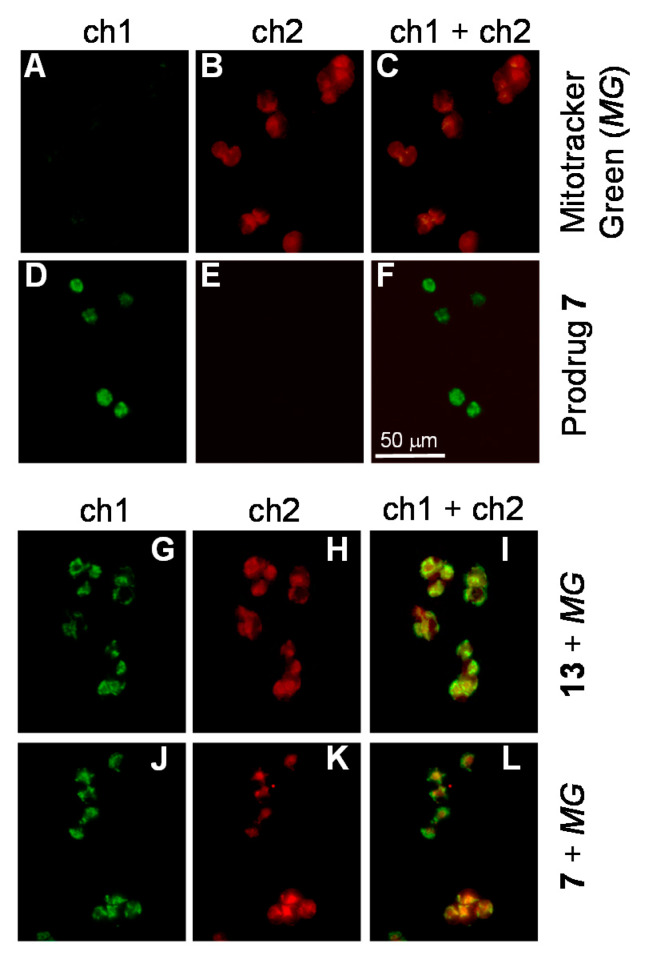
(**A**–**C**) Incubation of A2780 cells with Mitotracker green (MG, 100 nM) for 30 min and imaging the cells using channels 1 (ch1: excitation in the range of 450–490 nm and emission in the range of 500–550 nm) and 2 (ch2: excitation in the range of 538–563 nm and emission in the range of 570–640 nm). Mathematical sum of ch1 and ch2 is shown in inset **C**. (**D**–**F**) The same as **A**–**C**, except that MG was replaced with prodrug **7** (100 nM). (**G**–**I**) The same as **A**–**C**, except that MG was replaced with a mixture of control **13** (100 nM) and MG (1 eq). Pearson’s coefficient is 0.93; (**J**–**L**) The same as **A**–**C**, except that MG was replaced with a mixture of prodrug **7** (100 nM) and MG (1 eq). Pearson’s coefficient is 0.87.

These data indicate that the mitochondria-targeting moiety favorably affects the cellular uptake of NAAF-prodrugs. The uptake of prodrug **7** by BL-2 cells could be studied directly by making use of its intrinsic TMR^+^-derived fluorescence. We observed that the uptake is efficient and the loading of **7** in the cells correlates with its extracellular concentration (Figure 3B). Furthermore, we investigated the localization of prodrug **7** in human ovarian A2780 cells by using fluorescence microscopy. The latter cells were selected since they are adherent and, therefore, well compatible with fluorescence microscopy. We observed that prodrug **7** co-localizes with commercially available mitochondria-staining dye Mitotracker Green (MG, Figure 4). The same localization pattern was obtained for the control compound **13** lacking any cargo moiety. These data indicate that DLC’s (like TMR^+^) are suitable membrane carriers for the NAAF-prodrugs. 

Next, we studied the effects of new prodrugs and controls on the viability of selected cancer cells (BL-2, A2780, human prostate cancer DU-145 cells and human immortalized T lymphocyte Jurkat cells) as well as representative normal cells (human dermal fibroblasts adult, HDFa, Table 1). We observed that prodrug **5** exhibits overall stronger effects on the cell viability in all studied cell lines than non-targeted NAAF-prodrug **9** and unmodified ferrocene (**11**, negative control), but is less potent than unspecific positive control (Fe(HQ)_2_) (*p* < 0.001, *n* = 3 for all). For example, for BL-cells IC_50_ (48 h incubation) = 5 ± 1 μM for **5** versus 35 ± 2 μM for **9** (*p* < 0.001, *n* = 3). The same trend was observed for both shorter (24 h, *p* < 0.001, *n* = 3) and longer (96 h, *p* < 0.001, *n* = 3) incubation times (Table 1). However, another control compound **12**, which is an empty carrier Ph_3_P-R^+^, was even more cytotoxic, e.g., IC_50_(48 h incubation) = 0.5 ± 0.2 μM (*p* < 0.001, *n* = 3). The toxicity was retained towards the normal cell line HDFa (IC_50_ < 1 μM), whereas the effect of the hybrid prodrug **5** for HDFa cells was much more moderate (IC_50_ = 18 ± 5 μM). The higher cancer cell selectivity of **5** versus **12** can stem either from (a) the stronger NAAF-prodrug activation due to the higher ROS amount in mitochondria than in cytoplasm of cancer cells or (b) due to the favorable modulation of the high toxicity of Ph_3_P-R^+^ carrier by the NAAF-prodrug moiety.

We assume that, if (a) is valid, substitution of Ph_3_P-R^+^ in prodrug **5** for another efficient mitochondrial carrier (e.g., TMR^+^) should lead to the comparable effect on cytotoxicity and cancer specificity. However, if (b) is valid, replacement of PPh_3_P-R^+^ for a less toxic carrier (e.g., TMR^+^ in control **13**, IC_50_ = 35 ± 6 μM for BL-2 cells) should lead the strong attenuation of the effect. We believe that scenario (b) is operative, since prodrug **7** (derivative of TMR^+^ carrier) is nontoxic towards studied cancer cell lines (BL-2 and A2780). Interestingly, despite being more cytotoxic, prodrug **5** is a weaker ROS amplifier in BL-2 cells than non-targeted NAAF-prodrug **9** (*p* < 0.01, *n* = 3, Figure 3C). Since the intracellular ROS amount correlates with the efficacy of NAAF-prodrug activation [7,8,18], we conclude that the intracellular formation of the NAAF drug from prodrug **5** occurs with the lower yield than that from prodrug **9**. This is in agreement with suggestion (b) discussed above. 

### 2.3. The Mode of Action of Mitochondria-Targeting NAAF-Prodrug ***5***

**Figure 5:**
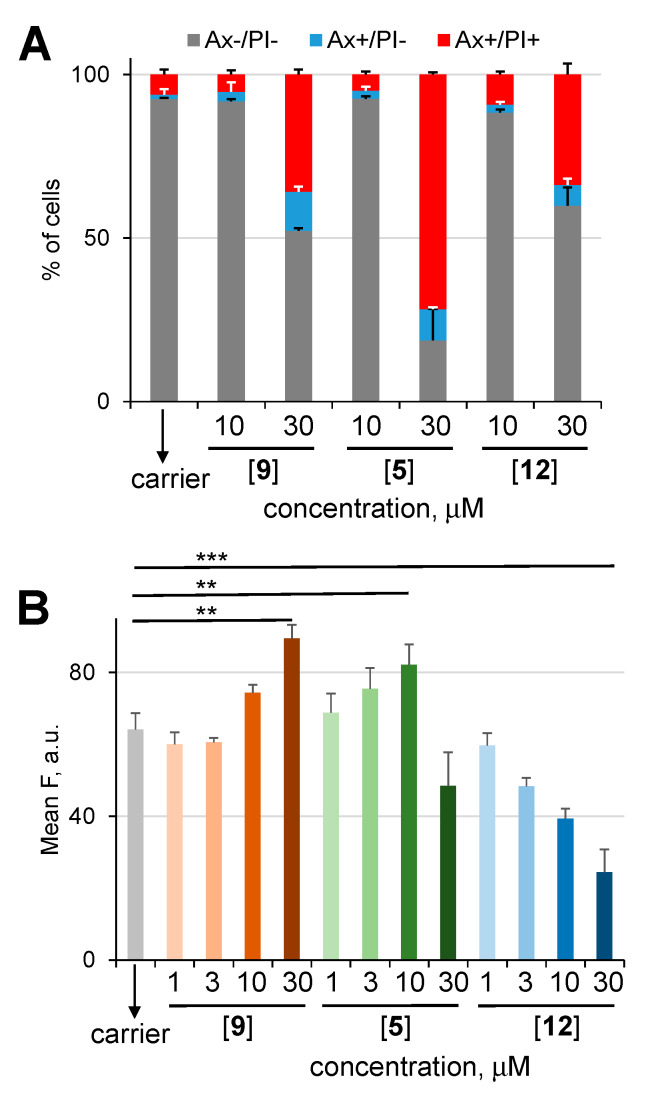
(**A**) Effect of prodrug **5** and controls **9** and **12** used at the indicated on the plot concentrations on the viability of Jurkat cells (incubation time: 48 h). The cells were stained and analyzed by flow cytometry as described in the SI. Annexin A5-FITC (Ax) and propidium iodide (PI) negative cells (indicated “Ax-/PI-“) were considered viable, “Ax+/PI-“—apoptotic and “Ax+/PI+” necrotic. (**B**) Effect of prodrug **5** and controls **9** and **12** used at the indicated on plot concentrations (incubation time: 48 h) on mitochondrial membrane potential of Jurkat cells. Fluorescence intensity corresponding to the potential-sensitive dye 1,1′,3,3,3′,3′-hexamethylindodicarbocyanine iodide (DiIC1(5)) is plotted on the Y-axis (λ_ex_ = 638 nm; λ_em_ = 655-695 nm). Student’s *t*-test: **—*p* < 0.01; ***—*p* < 0.001.

To get a deeper insight into the mode of action of the more active prodrug **5**, we first determined via which mechanism this compound induces the death of Jurkat cells. The latter cells were selected as representative cancer cells (Figure 5A). Prodrug **5** as well as controls **9** (non-targeted NAAF-prodrug) and **12** (an empty carrier) kill the cells mostly via necrosis (red bars, Figure 5A) and apoptosis (blue bars, Figure 5A) with the former cell death phenotype being more prominent.

We could not distinguish between direct necrosis and apoptosis followed by secondary necrosis. Furthermore, at nontoxic concentrations ≤ 10 μM prodrug **5** induces the significant increase of the mitochondrial potential (green bars, Figure 5B) that has been previously observed for other targeted and non-targeted NAAF-prodrugs (e.g., **9**, orange bars, Figure 5B). In contrast, the empty carrier **12** reduces the mitochondrial potential at all tested concentrations (blue bars, Figure 5B). Thus, conjugation of the NAAF-moiety to the carrier attenuates its negative effect on the mitochondria membrane potential that leads to its lower toxicity. 

**Figure 6:**
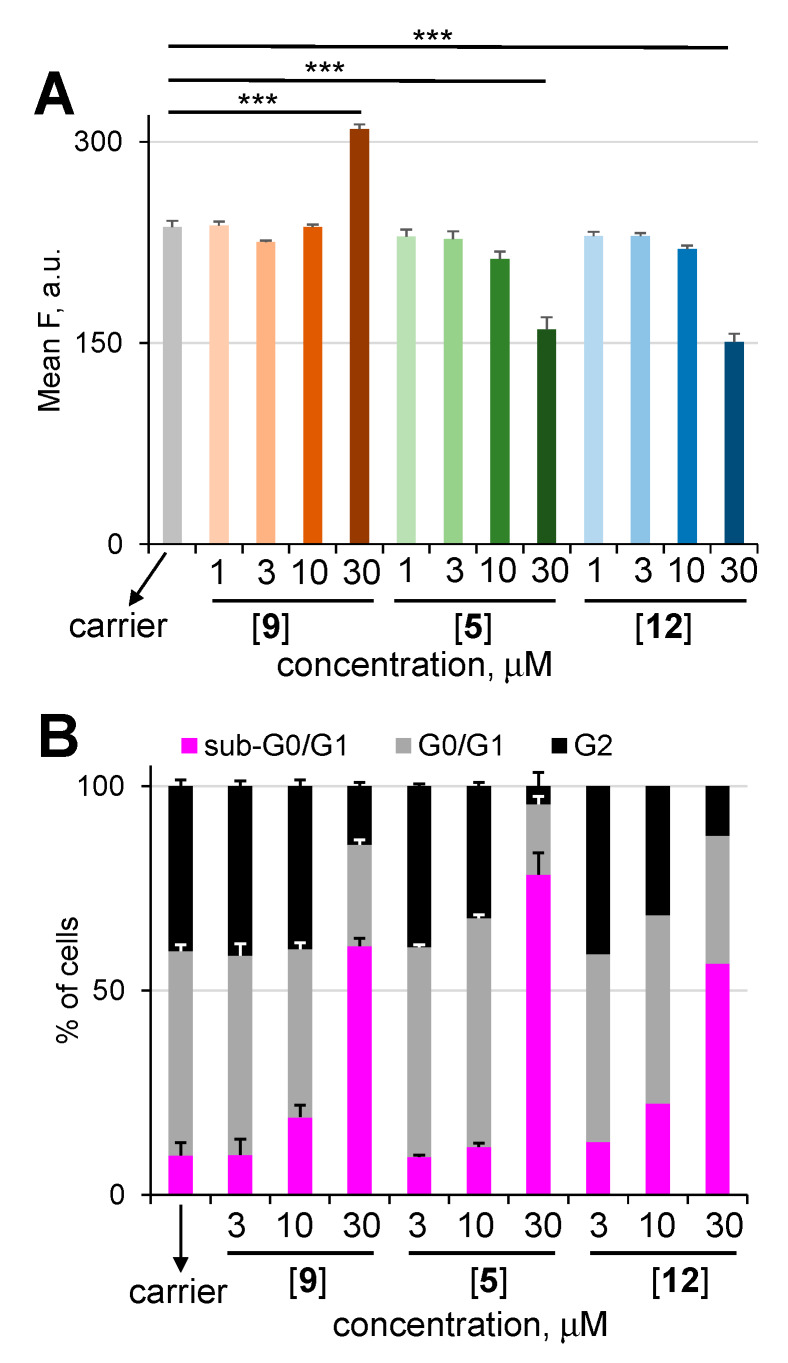
(**A**) Effect of prodrug **5** and controls **9** and **12** used at the indicated on the plot concentrations on the intracellular glutathione (GSH) content of viable (Ax-/PI-) Jurkat cells (incubation time: 48 h). Fluorescence intensity corresponding to GSH-sensitive dye monobromobimane is plotted on the OY-axis (λ_ex_ = 405 nm; λ_em_ = 390–470 nm). (**B**) Effect of prodrug **5** and controls **9** and **12** used at the indicated-on plot concentrations (incubation time: 48 h) on cell cycle of Jurkat cells. Student’s *t*-test: ***—*p* < 0.001.

Finally, at the concentrations causing no cell death prodrug **5** does not affect the intracellular concentration of glutathione (Figure 6A) and cell cycle (Figure 6B). In contrast, typical NAAF-prodrugs induce upregulation of intracellular GSH to counteract elevated ROS and cell cycle arrest in the G0/G1 phase [18]. 

## 3. Materials and Methods

Commercially available chemicals of the best quality from Sigma-Aldrich (Schnelldorf, Germany) and Alfa-Aesar (Kandel, Germany) were obtained and used without purification. NMR spectra were acquired on a Bruker Avance 300 (Ettlingen, Germany) or Bruker Avance 400 (Ettlingen, Germany). ESI mass spectra were recorded on a Bruker ESI MicroTOF II (Bremen, Germany) or Bruker maXis 4G mass spectrometers (Bremen, Germany). C/H/N elemental analysis was performed in the microanalytical laboratory of the Department Chemistry and Pharmacy, Organic Chemistry Chairs 1 and 2 of the Friedrich-Alexander-University of Erlangen-Nuremberg. UV-visible spectra were measured on a Cary 100 UV-visible spectrophotometer (Agilent Technologies, Frankfurt am Main, Germany) by using either quartz glass cuvettes (Hellma GmbH, Müllheim, Germany) with a sample volume of 1 mL or micro-cuvettes with a sample volume of 100 µL (BRAND GmbH, Wertheim, Germany). Fluorescence spectra were acquired on a Varian Cary Eclipse fluorescence spectrophotometer using fluorescence cuvettes (Hellma GmbH, Müllheim, Germany) with a sample volume of 1 mL. The fluorescence of live cells was quantified by using a Guava easyCyteTM 6-2L Flow cytometer from Merck Millipore. The data were processed using the inCyteTM software package (Merck Millipore, Darmstadt, Germany) and the ModFIT LTTM software (Verity Software House, Topsham, ME, USA). The fluorescence images of live cells were acquired by using a Zeiss Axio Vert.A1 microscope (Jena, Germany) using 40x/1.30 objective (oil DIC) and the following filter set: 450-490/500-550 nm for the detection of MitoTracker Green (channel 1: abbreviated ch1 in the main text of the paper); excitation/emission 538–563/570–640 nm for the detection of prodrug **7** and control 13 (channel 2: abbreviated ch2 in the main text of the paper). Statistical analysis of the data was conducted by using Student’s *t*-test. Two data sets were considered significantly different from each other for *p* < 0.05.

Syntheses of new prodrugs and control compounds as well as protocols of all biological assays used in this study are described in detail in the SI to this article.

### 3.1. Determination of Solubility of Prodrugs ***5*** and ***7*** in Aqueous Buffers 

Prodrugs **5**, **7** and **9** (known control) were dissolved in DMSO at concentrations of 15, 10, 5, 2, 1.5, 1, and 0.2 mM. These solutions (10 μL) were transferred to quartz cuvettes containing 990 μL of either Dulbecco’s phosphate-buffered saline (DPBS) or Roswell Park Memorial Institute (RPMI) medium containing 20% of fetal bovine serum (FBS) to obtain solutions (or suspensions) with final concentrations of prodrugs—150, 100, 50, 20, 15, 10, and 2 μM. The absorbance at 790 nm of the resulting solutions/suspensions was measured and plotted as a function of the concentration of prodrugs. Mixtures absorbing <0.04 (A_790 nm_) for FBS and 0.034 (A_790 nm_) for DPBS appeared transparent and were considered as true solutions. Using these data, we determined solubility limits for prodrugs and controls (Table S1).

### 3.2. Monitoring Generation of Reactive Oxygen Species (ROS) in the Presence of Either Prodrugs or Control Compounds in Cell Free Settings 

2′,7′-Dichlorofluorescin diacetate (DCFH-DA, 4.9 mg) was dissolved in 100 µL DMF and mixed with 0.1 M NaOH (900 µL). This mixture was incubated on a shaker for 30 min at 25 °C in the dark to obtain 10 mM 2′,7′-dichlorofluorescin (DCFH) stock solution. Next, 3-(*N*-morpholino)propanesulfonic acid buffer (MOPS buffer 100 mM, pH: 7.4) containing *N*,*N*,*N*’,*N*’-ethylenediaminetetraacetic acid (EDTA, 10 mM), glutathione (GSH, 5 mM) and H_2_O_2_ (10 mM) was prepared. Finally, DCFH was diluted in this buffer to 10 µM as the final concentration. Monitoring of the fluorescence was started (λ_ex_: 501 nm, λ_em_: 525 nm). After 5 min, the compound was added to have a final concentration of 50 µM. DMF and FeSO_4_ in DMF were used as controls. The measurement was conducted for 300 min (Table S2, Figure 1A).

### 3.3. Monitoring Activation of Prodrug ***7*** in the Presence of H_2_O_2_ by Fluorescence Spectroscopy

Prodrug **7** (10 µL, 50 µM in DMSO) was added into phosphate buffered saline (1000 µL, PBS: 10 mM PO_4_^3−^ and 150 mM NaCl, pH: 7.0, final concentration 0.5 µM). After the initial scan of the fluorescence at 37 °C (λ_ex_: 551 nm, λ_em_: 576 nm), H_2_O_2_ was added (10 µL, final concentration: 50 mM) into the mixture and changes of the fluorescence intensity was monitored for the next 120 min. As a control, the fluorescence change in the same mixture lacking H_2_O_2_ was also monitored (Figure 1B). 

### 3.4. Monitoring of H_2_O_2_-Induced Activation of Prodrug ***5*** by Using ESI Mass Spectrometry 

A freshly prepared solution of prodrug **5** (10 µL, 4 mM in DMF) was diluted with CH_3_CN (100 µL). The resulting solution was mixed with water (886 µL) followed by the addition of either H_2_O_2_ (4 µL, 30% (*w*/*w*) in H_2_O) or water (4 µL, negative control). These solutions were kept at 22 °C for 5 min. Then they were diluted with CH_3_CN (1 mL) and the resulting probes were analyzed by high resolution ESI-TOF mass spectrometry in the positive mode (Figure 2). The following reaction products could be identified (Figure S10).

### 3.5. Cells and Cell Cultures 

Burkitt lymphoma cell line (BL-2) and Jurkat cells were obtained from DSMZ (Germany). Human ovarian cell line (A2780) was obtained from Sigma-Aldrich. The human prostate cell line (DU-145) was kindly provided by the lab of Prof. Dr. Olaf Prante (Clinic of Nuclear Medicine, Friedrich-Alexander-University Hospital Erlangen) and Human Dermal Fibroblast adult (HDFa) cell line was kindly provided by the laboratory of Prof. Dr. C. Alexiou (SEON, Friedrich-Alexander-University Hospital Erlangen). BL-2 cells were grown in RPMI 1640 medium supplemented with 20% FBS, 1% L-glutamine, and 1% penicillin/streptomycin. A2780 cells were grown in RPMI 1640 medium supplemented with 10% FBS, 1% L-glutamine, and 1% penicillin/streptomycin. DU-145 and NHDF cell lines were grown Dulbecco’s modified eagle medium red (DMEM, Biochrom GmbH, Germany) supplemented with 10% FBS, 1% L-glutamine, and 1% penicillin/streptomycin. Jurkat cells were cultivated in RPMI 1640 medium supplemented with 10% fetal bovine serum (FBS), and 1% glutamine. Suspension cells were grown to (0.5–1.5) × 10^6^ cells/mL and diluted as required. Adherent cells were cultivated to 80–90% confluence and detached from the flask by using trypsin/EDTA solution (0.025%/ 0.01%, *w*/*v*, Biochrom GmbH, Germany) in PBS. 

### 3.6. Determination of the Viability of Adherent Cells (A2780, DU-145 and HDFa) 

Adherent A2780, DU-145 and NHDF cells were grown as mentioned before. Then, the cells were resuspended in the RPMI 1640 (DMEM for HDFa cell line) medium containing 5% FBS (for 24- and 48 h assays) or 10% FBS (for 96 h assays) 1% L-glutamine, and 1% penicillin/streptomycin. This suspension was spread in the wells of a 96-well microtiter plate. Every well in the plates contained 25,000 cells (exceptions: 12,500 cells for the 96h-assay and 10,000 cells for experiments with HDFa cell line) per well per 100 μL and was left at 37 °C in the chamber filled with CO_2_ (5%) overnight. Stock solutions of prodrugs of different concentrations (1 μL, solvent DMSO) were added to the wells and incubated for 24–96 h. Four experiments were conducted for each concentration of the prodrug. Finally, 3-(4,5-dimethylthiazol-2-yl)-2,5-diphenyltetrazolium bromide (MTT; 20 μL of the solution prepared by dissolving MTT (5 mg) in PBS buffer (1 mL)) was added to each well, incubated for 3 h, treated with sodium dodecyl sulfate (SDS) solution (90 μL, 10% solution in 0.01 M aqueous HCl), and incubated overnight. Then the intensity of absorbance at 590 nm was measured (MTT is converted to blue dye (λ_max_ = 590 nm) in live cells). The absorbance at 690 nm was taken as a baseline value. These data were applied to calculate the relative number of viable cells.

### 3.7. Determination of the Viability of Suspension BL-2 Cells

The suspension of BL-2 cells in the RPMI 1640 medium containing 5% FBS (for 24- and 48 h assays) or 10% FBS (for 96 h assays) 1% L-glutamine, and 1% penicillin/streptomycin was spread in the wells of a 96-well microtiter plate. Every plate contained 50,000 cells (with the exception of the 96h-experiment, for which 25,000 cells were used) per well per 100 μL. Stock solutions of prodrugs of different concentrations (1 μL, solvent DMSO) were added to the wells and incubated for 24–96 h. Four experiments were conducted for each concentration of the prodrug. Finally, 3-(4,5-dimethylthiazol-2-yl)-2,5-diphenyltetrazolium bromide (MTT; 20 μL of the solution prepared by dissolving MTT (5 mg) in PBS buffer (1 mL)) was added to each well, incubated for 3 h, treated with sodium dodecyl sulfate (SDS) solution (90 μL, 10% solution in 0.01 M aqueous HCl), and incubated overnight. Then the intensity of absorbance at 590 nm was measured (MTT is converted to blue dye (λ_max_ = 590 nm) in live cells). The absorbance at 690 nm was taken as a baseline value. These data were applied to calculate the relative number of viable cells. All obtained data are summarized in Table 1.

### 3.8. Determination of Prodrug 5 Uptake by BL-2 Cells by Quantification of Intracellular Boron Amounts Using the Curcumin-Assay

BL-2 cells were grown as mentioned before. The suspension of the cells in the RPMI 1640 medium with 5% FBS 1% L-glutamine, and 1% penicillin/streptomycin containing 10^6^ cells/mL was spread in the wells of a 6 well microtiter plates. Solutions of prodrugs (30 μL per well, solvent DMSO, each well contained 3 mL cell suspension) were added to the suspensions and incubated for 1 h. The final concentration of the prodrugs in the suspensions was 1 μM. This low concentration was used to avoid cell death, since some prodrugs in this series (e.g., prodrug 5) are very toxic. The suspensions from 12 wells treated at the same condition were combined, the combined suspensions were washed three times with ice-cold PBS (3 × 5 mL), treated with concentrated H_2_O_2_ solution (200 μL, 1 M) for 30 min, and all volatiles were removed by lyophilization. Dry, lysed cells were washed with water (200 μL), and the aqueous solution obtained was acidified with HCl (400 μL, 0.1 M). Then this solution was extracted with 2-ethyl-1,3-hexanediol (160 μL, 10% in CHCl_3_, *v*/*v*), and a portion of the organic phase obtained (90 μL) was mixed with H_2_SO_4_/CH_3_CO_2_H (400 μL, 1/1, *v*/*v*). Curcumin solution in methyl isobutyl ketone (250 μL, 2 mg in 1 mL of the solvent) was added and allowed to react for 24 h. The reaction was quenched by addition of water (1 mL). The light absorbance at 550 and 780 nm of the organic phase was measured. The former value corresponds to absorbance of a curcumin–boron complex, whereas the second one is taken as a baseline. The baseline corrected absorbance at 550 nm (∆A = A(550 nm) − A(780 nm)) was proportional to the concentration of boron in the mixture (Table S3).

### 3.9. Determination of Prodrug 7 Uptake by BL-2 Cells by Quantification the Fluorescence Derived from 7 Using Flow Cytometry

The cells were centrifuged (5 min; 1000 rpm), washed with DPBS (2 × 10 mL) and concentrated at cell density of 10^6^ cells/mL in opti-MEM. The cell suspension was split into portions as 200 µL in each. Prodrug 7 (dissolved in DMSO) was added into these suspensions (2 µL) to obtain final concentrations of prodrugs 10 and 25 μM. As a negative control pure DMSO (2 µL) was added to the cell suspension. After 120 min of incubation in the dark chamber filled with 5% CO_2_ at 37 °C, the mean fluorescence of live cells (λ_ex_ = 488 nm, λ_em_ = 530 nm) in the suspensions was determined by using the flow cytometer (Figure 3B).

### 3.10. Determination of ROS-Amplification in BL-2 Cells in the Presence of Prodrugs 

The cells were centrifuged (5 min; 1000 rpm), washed with DPBS (2 × 10 mL) and the suspension at cell density of 10^6^ cells/mL in DPBS was prepared. 5(6-)chloromethyl-2′,7′-dichlorodihydrofluorescein diacetate (CM-DCFH-DA) in DMSO (8.7 µL, stock solution 10 mM) was added into 8.7 mL of cell solution to have 10 µM final concentration. The cells were incubated in the dark chamber supplemented by 5% CO_2_ at 37 °C for 15 min. Then, the cells were centrifuged, washed with DPBS (1× 10 mL) and resuspended in opti-MEM medium by maintaining the cell concentration at 10^6^ cells/mL. The cell suspension was split into portions as 200 µL in each. The compounds (prodrug **5**, **7**, **9**) and the negative and positive controls (Ferrocene (11) and Fe(8HQ)_2_ complex, respectively), as well as the DMSO were added from the stock solutions (2 µL). After 120 min of incubation in the dark chamber filled with 5% CO_2_ at 37 °C, the mean fluorescence of live cells (λ_ex_ = 488 nm, λ_em_ = 530 nm) in the suspensions was determined by using the flow cytometer. All samples were applied in triplicates within the assay and the results were calculated based on three independent assays. Prodrug **7** contributes to the fluorescence signal derived from CM-DCFH-DA (λ_ex_ = 488 nm, λ_em_ = 530 nm). This background signal was quantified by doing the same experiment as described above, but in the absence of CM-DCFH-DA (Figure 3B). Then we subtracted this background signal from that obtained in the presence of CM-DCFH-DA to obtain the corrected ROS-mediated fluorescence increase of CM-DCFH-DA probe (Figure 3C). 

Co-localization of prodrug 7 (or control 13) and MitoTracker Green probe in a representative cancer cell (A2780) by using fluorescence microscopy A2780 cells were seeded on a 35 mm imaging dish (µ-Dish 35 mm, high, ibidi GmbH, Germany) at a cell density of 80 cells/µL one day before the experiment in 2000 µL RPMI 1640 medium containing 5% FBS, 1% L-glutamine and 1% penicillin/streptomycin. On the next day, the cells were washed with DPBS (2 × 2 mL). A fresh portion of RPMI 1640 medium (2 mL) was added. Solutions of MitoTracker Green (20 µL, 10 µM in DMSO, Thermo Fisher Scientific, Freiburg im Breisgau, Germany), prodrug 7 (or control 13) in DMSO (20 μL, 10 µM) were added into separate dishes, yielding the final concentration of reagents—100 nM. In another experiment, a mixture of 7 and MitoTracker Green (or 13 and MitoTracker Green) were added to the cells. The cells were kept for 30 min in the incubator at 37 °C containing 5% CO_2_. The cells were washed with DPBS (2 × 2 mL) and the fresh medium (2 mL) was added. The fluorescence images were taken with a Zeiss Axio Vert.A1 and filter set: excitation/emission 538–563/570–640 nm for the detection of 7 and 13 and 450–490/500–550 nm for the detection of MitoTracker Green. Objective: 40x/1.30. Oil DIC (Figure 3).

### 3.11. Experiments with Jurkat Cells 

Jurkat cells were counted in MUSE Cell Analyzer using MUSE^®^ Count &Viability Assay Kit (Merck-Millipore, Billerica, MA, USA) and adjusted to a density of 1.0 × 10^5^/mL in RPMI 1640 medium (with 10% FCS and 1% glutamine). A total of 9000 cells per well per 90 μL were seeded into 96 well culture plates. The 50 mM prodrug stock solutions were diluted to 300, 100, 30, and 10 μM in cell culture medium and 10 μL of the dilutions was pipetted to the cells, receiving final test concentrations of 30, 10, 3, and 1 μM. Every concentration was tested in triplicates. Cells with corresponding amounts of DMSO served as negative controls. After 48 h cells were mixed and 25 μL of the cell suspension was incubated with 200 μL of freshly prepared staining solution, consisting of 1 μl Annexin A5-Fitc, 0.4 μL 1,1′,3,3,3′,3′-hexamethylindodicarbocyanine iodide (DiIC1(5)) (both from Thermo Fisher Scientific, Waltham, MA, USA), 66.6 ng propidiumiodide (PI) and 33 μM monobromobimane (MBB, both from Sigma-Aldrich, Taufkirchen, Germany) per 1 mL Ringer’s solution (Fresenius Kabi AG, Bad Homburg, Germany). Cells were incubated for 20 min at 4 °C and then analyzed in Gallios cytofluorometer™ (Beckman Coulter, Fullerton, CA, USA). Fitc and propidium iodide were excited at 488 nm; Fitc fluorescence was recorded on the FL1 sensor (525/38 nm band pass, BP) and propidium iodide fluorescence was detected on FL3 sensor (620/30 nm BP), DiIC1(5) fluorescence was excited at 638 nm and detected on FL6 sensor at 675/20 nm. Monobromobimane fluorescence was excited at 405 nm and detected on FL9 sensor at 430/40 nm. To eliminate any fluorescence bleed-through, electronic compensation was used. Data were analyzed employing Kaluza™ software Version 1.2 (Beckman Coulter, Fullerton, CA, USA) and processed in Microsoft Excel. Cells negative for Annexin A5- Fitc and propidium iodide were considered viable. IC_50_ values were determined by fitting the % values of Ax-PI- cells versus drug concentration with a sigmoidal curve using GraphPad Prism software. Reduced glutathione (an intracellular thiol) can be detected by monobromobimane, a dye that becomes fluorescent after non-enzymatic binding to thiols. For analysis of the amount of mitochondrial membrane potential or reduced glutathione, the mean fluorescence index (MFI) was determined from viable cells by gating on Ax-PI-events. 

## 4. Conclusions

We successfully prepared two hybrids **5** and **7** containing an *N*-alkylaminoferrocene (NAAF)-based prodrug attached to representative mitochondria-targeting moieties—either triphenylphoshonium cation or *N*,*N*,*N*′,*N*′-tetramethylrhodamine correspondingly. We confirmed that these hybrids are efficiently uptaken by cells, and on the example of **7** we confirmed that they are localized in the mitochondria of cancer cells. Based on the effects of the prodrugs on the viability of representative cell lines and mechanistic studies of their mode of action, including determination of the mechanism of cell death, effects of the hybrids on intracellular ROS and GSH, mitochondrial potential and cell cycle, we concluded that the activation of the NAAF-prodrug is not facilitated by its targeting to mitochondria. Possibly, ROS released by mitochondria are quickly scavenged by intracellular antioxidants and are not available for activation of the ROS-responsive prodrugs. Thus, hybrid **5** does not act as a typical ROS amplifier. Its mode of action relies off the mixed effect of weak ROS generation by the NAAF-part and modulation of membrane potential by the Ph_3_P-R^+^ carrier part. Despite the unexpected mode of action, hybrid **5** exhibits favorable properties including substantially higher cytotoxicity towards cancer cells than that of non-targeting NAAF-prodrugs as well as lower cytotoxicity towards normal HDFa cells with respect to the empty carrier **12**. 

The cancer cell specificity observed in cell-based assays may justify in vivo studies of **5** in the future. However, this experiment will become possible only after a suitable formulation of poorly water-soluble prodrug **5** is found. Since NAAF-prodrugs are only moderately stable in blood [23], intraperitoneal (i.p.) injection remains the method of choice for administration of prodrug **5** in vivo, as reported for known NAAF-prodrugs [18].

## Figures and Tables

**Table 1:** Effects of prodrugs and control compounds on the viability of selected cancer and normal cells lines expressed as half-maximal inhibitory concentration (IC_50_, μM).

Prodrug or Control	IC_50_/BL-2 Cells *^i^* (μM)		
	24 h *^ii^*	48 h *^ii^*	96 h *^ii^*
**5**	5 ± 1	5 ± 1	8 ± 1
**12**	2 ± 1	0.5 ± 0.2	0.3 ± 0.1
**9**	37 ± 2	35 ± 2	33 ± 2
**7**	−	>25	−
**13**	−	35 ± 6	−
**11**	>50	>50	>50
Fe(HQ)_2_ *^iii^*	3 ± 1	3 ± 1	3 ± 1
	IC_50_/A2780 cells *^i^* (μM)		
	24 h *^ii^*	48 h *^ii^*	96 h *^ii^*
**5**	10 ± 3	5 ± 2	5 ± 2
**9**	42 ± 3	32 ± 3	30 ± 5
**7**	−	>25	−
**13**	−	>50	−
**11**	>50	>50	>50
Fe(HQ)_2_	3 ± 1	3 ± 1	3 ± 1
	IC_50_/DU-145 cells *^i^* (μM)		
	24 h *^ii^*	48 h *^ii^*	96 h *^ii^*
**5**	20 ± 3	10 ± 2	10 ± 2
**9**	>50	47 ± 2	35 ± 3
**11**	>50	>50	>50
Fe(HQ)_2_	3 ± 1	3 ± 1	3 ± 1
	IC_50_ (μM)		
	Jurkat cells *^i^*		HDFa cells *^i^*48 h *^ii^*
	24 h *^ii^*	48 h *^ii^*	
**5**	20 ± 3	20 ± 2	18 ± 5
**12**	−	−	<1
**9**	30 ± 3	30 ± 4	>50
**11**	>50	>50	−

*^i^* Burkitt lymphoma cell line: BL-2; Human ovarian cell line: A2780; human prostate cell line: DU-145; human immortalized T lymphocyte cells: Jurkat; human dermal fibroblasts, adult: HDFa. *^ii^* Times of incubation of the prodrugs and controls with corresponding cells. *^iii^* Fe(HQ)_2_: 1 to 2 mixture of FeCl_3_ and 8-hydroxyquinoline.

## Supplementary Materials

The following are available online, Figure S1: ^1^H NMR spectrum of prodrug **5** in DMSO-*d*_6_; Figure S2: ^13^C NMR spectrum of prodrug **5** in DMSO-*d*_6_; Figure S3: ^31^P NMR spectrum of prodrug **5** in DMSO-*d*_6_; Figure S4: High resolution ESI-TOF mass spectrum of prodrug **5**: upper plot—experimental spectrum; bottom plot—theoretical spectrum; Figure S5: ^13^C NMR spectrum of intermediate **6** in CDCl_3_; Figure S6: ^1^H NMR spectrum of intermediate **6** in CDCl_3_; Figure S7: High resolution ESI-TOF mass spectrum of intermediate **6**: upper plot—full experimental spectrum; bottom plot—experimental spectrum zoomed for MP region; Figure S8: ^1^H NMR spectrum of 5-(trimethylsilyl)ethynyl-tetramethylrhodamine in CDCl_3_. Impurities are marked with asterisk; Figure S9: DEPTQ spectrum of 5-(trimethylsilyl)ethynyltetramethylrhodamine in CDCl_3_. Impurities are marked with asterisk; Figure S10: ^1^H NMR spectrum of intermediate **13** in CDCl_3_. Impurities are marked with asterisk; Figure S11: DEPTQ spectrum of intermediate **13** in CDCl_3_; Figure S12: ^13^C NMR spectrum of intermediate **13** in CDCl_3_; Figure S13: ^1^H NMR spectrum of prodrug **7** in DMSO-*d*_6_. Impurities are marked with asterisk; Figure S14: High resolution ESI-TOF mass spectrum of prodrug **7**: upper plot—theoretical spectrum; bottom plot—experimental spectrum; Figure S15: Zoomed in regions of the mass spectrum shown Figure 2B; Table S1. Solubility of prodrugs in aqueous solutions; Table S2. Initial rates of DCFH oxidation in the presence of H_2_O_2_ and prodrugs/controls; Table S3. Uptake of prodrug **5** by BL-2 cells.
