# Supplementary material for: N-Alkylaminoferrocene-Based Prodrugs Targeting Mitochondria of Cancer Cells
Source: Molecules. 2020 May 29;25(11):2545. doi: 10.3390/molecules25112545 (PMC7321169; doi:10.3390/molecules25112545)
Supplement: Supplementary file 1 [file molecules-25-02545-s001.pdf]

N-Alkylaminoferrocene-Based Prodrugs Targeting Mitochondria of Cancer Cells

Viktor Reshetnikov, Hülya Gizem Özkan, Steffen Daum, Christina Janko, Christoph Alexiou, Caroline Sauer, Markus R. Heinrich, Andriy Mokhir*

Table of content

Synthesis	S2-S16
Additional data not included in the main text of the paper	S16-S18
References	S18

Synthesis Control prodrug **9** (Scheme 2, main text of the paper) was synthesized as described elsewhere.[1] Control **12** (Scheme 2, main text of the paper) was synthesized according to the known protocol.[2]

<u>Synthesis of prodrug **5**</u>

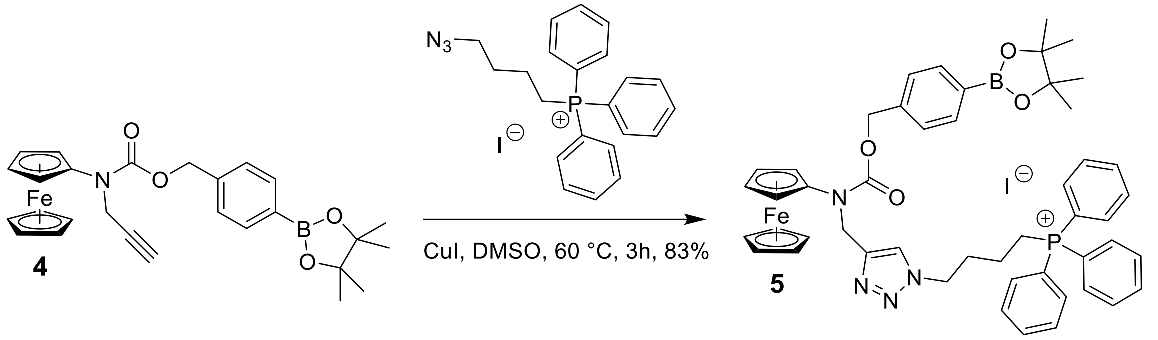

Starting material **4**[3] (190 mg, 380 µmol), 4-azidobutyl)triphenylphosphonium iodide[4] (223 mg, 457 µmol) and copper iodide (14.5 mg, 76.1 µmol) were dissolved in dimethylsulfoxide (DMSO, 3 mL) and stirred under nitrogen atmosphere at 50° C for 3 h. After cooling to 22 °C, water (6 mL) was slowly added under gentle shaking. The resulting precipitate was centrifuged, the supernatant removed and water (3 mL) added again. The mixture was homogenized and centrifuged, the supernatant was removed. After drying under high vacuum, a yellowish solid (310 mg, 0.314 mmol, 83%) was obtained. ^{1}H-NMR (400 MHz, DMSO): δ = 7.97 – 7.83 (m, 4H), 7.83 – 7.70 (m, 12H), 7.67 (d, J = 7.8 Hz, 2H), 7.37 (d, J = 7.5 Hz, 2H), 5.18 (s, 2H), 4.91 (s, 2H), 4.53 (s, 2H), 4.42 (t, J = 6.6 Hz, 2H), 4.09 (s, 5H), 4.02 (s, 2H), 3.72 – 3.55 (m, 2H), 2.09 – 1.89 (m, 2H), 1.61 – 1.42 (m, 2H), 1.28 (s, 12H) ppm. ^{13}C-NMR (101 MHz, DMSO): δ = 153.68, 144.17, 139.73, 135.00, 134.58, 133.60, 133.50, 130.37, 130.25, 127.14, 123.33, 118.73, 117.87, 83.76, 68.97, 68.80, 66.82, 64.27, 62.52, 48.14, 40.43, 30.38, 30.21, 24.70, 19.95, 19.44, 18.82 ppm. ^{31}P NMR (162 MHz, DMSO): δ = 25.08 ppm. High resolution ESI-mass spectrometry (positive mode): calcd. for $C_{49}H_{53}BFeN_4O_4P$: 859.3241; found m/z: 859.3263. Elemental analysis: calcd (%) for $C_{49}H_{53}BFeN_4O_4PI$: C 59.66, H 5.42, N 5.68; found: C 59.67, H 5.34, N 5,51.

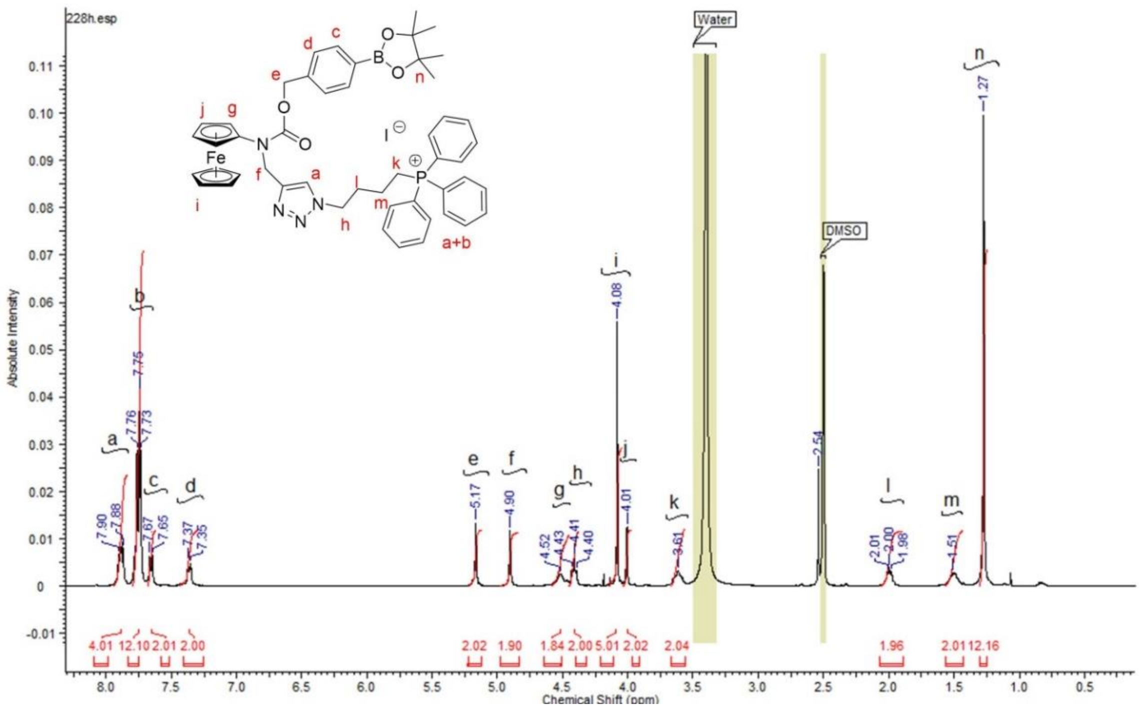

Figure S1. ^{1}H NMR spectrum of prodrug **5** in DMSO-d6.

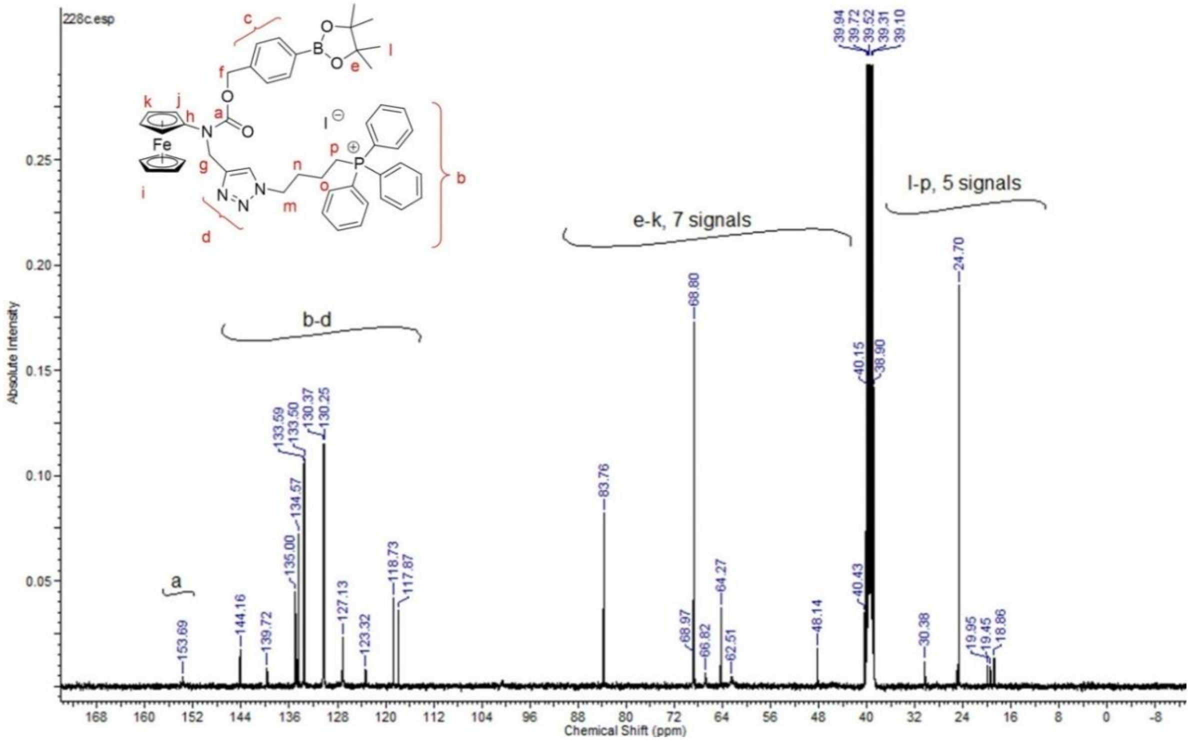

Figure S2. ^{13}C NMR spectrum of prodrug **5** in DMSO-d6.

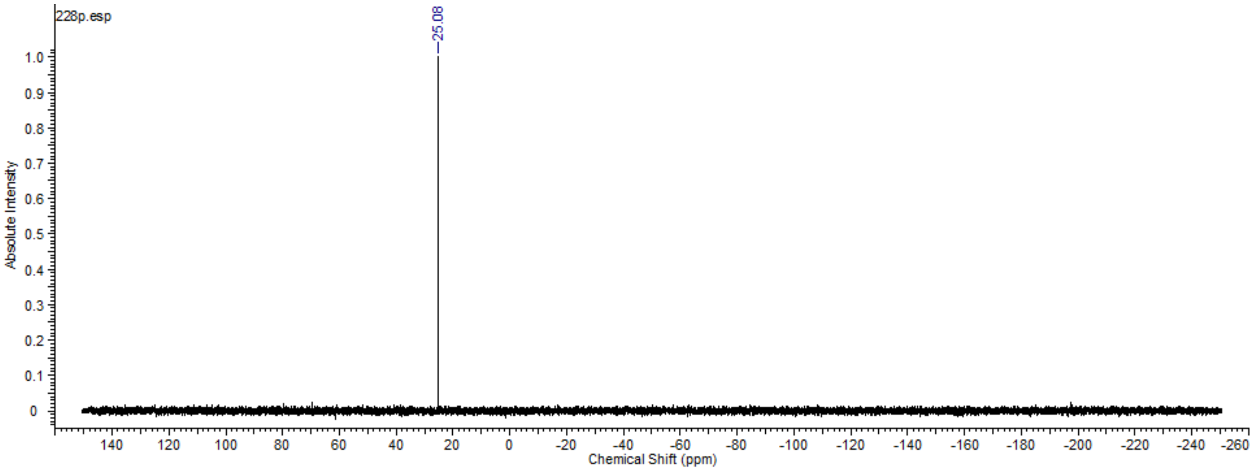

Figure S3. ^{31}P NMR spectrum of prodrug **5** in DMSO-d6.

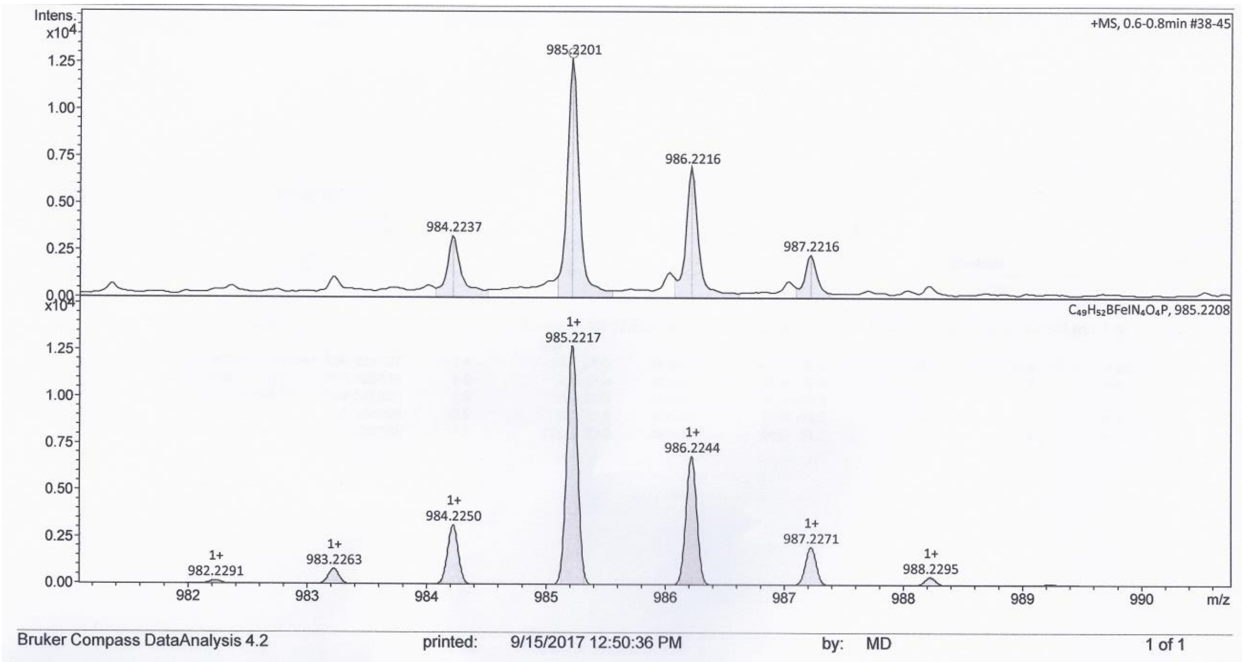

Figure S4. High resolution ESI-TOF mass spectrum of prodrug **5**: upper plot – experimental spectrum; bottom plot – theoretical spectrum.

Synthesis of intermediate **6**

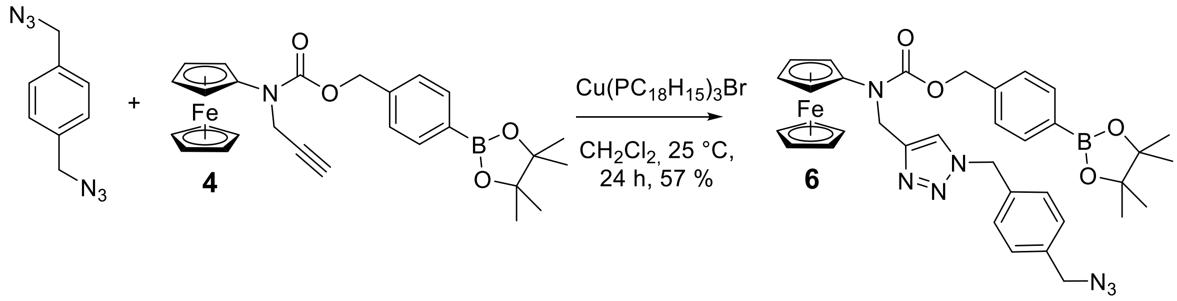

Compound **4** (2.00 g, 4.01 mmol), 1,4-bis(azidomethyl)benzene (3.02 g, 16.03 mmol) and bromotris(triphenylphosphine)copper(I) (745.46 mg, 801.30 µmol) were dissolved in CH_2Cl_2 (50 mL) and stirred under nitrogen atmosphere at 25° C for 24 h, followed by evaporation of the volatiles *in vacuo* (10 mbar). The crude product (**6**) was purified by column chromatography (silica gel, CH_2Cl_2/acetone 95/5, v/v, R_f = 0.50). Product **6** was obtained as an orange solid (1.358 g, 1.97 mmol, yield: 57 %). ^{1}H-NMR (300 MHz, $CDCl_3$): δ = 7.80-7.73 (m., 2H), 4.32-4.14 (m., 7H) 5.37 (br. s., 2H), 5.11-5.04 (m., 4H) 4.45-3.97 (m., 9H) 1.32 (s., 12H). ^{13}C-NMR (75 MHz, $CDCl_3$): δ = 145.97, 139.00, 138.97, 136.08, 134.99, 134.46, 128.81, 128.51, 127.88, 127.38, 122.71, 83.89, 77.20, 69.32, 67.51, 64.82, 62.60, 54.16, 53.76, 53.38, 24.84. High resolution ESI-mass spectrometry (positive mode): calcd. for 687.2428; found m/z: 687.2416. Elemental analysis: calcd (%) for $C_{35}H_{38}BFeN_7O_4$: C 61.16, H 5.57, N 14.26; found: C 61.17, H 5.54, N 13,85.

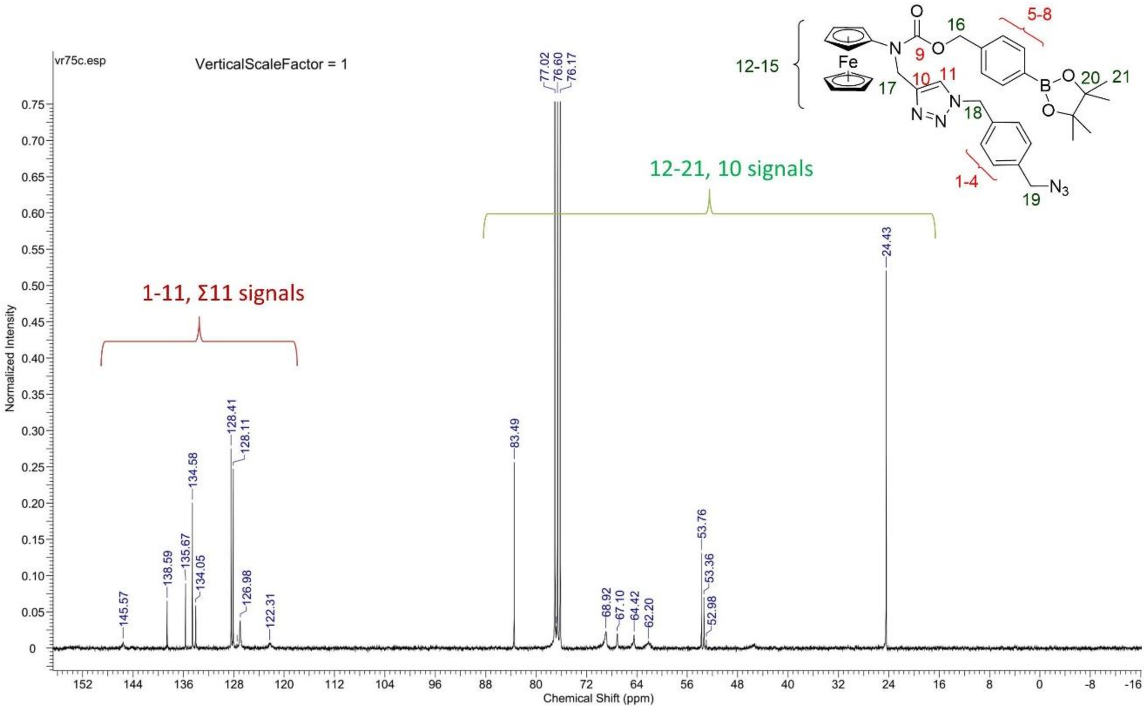

Figure S5. ^{13}C NMR spectrum of intermediate **6** in $CDCl_3$.

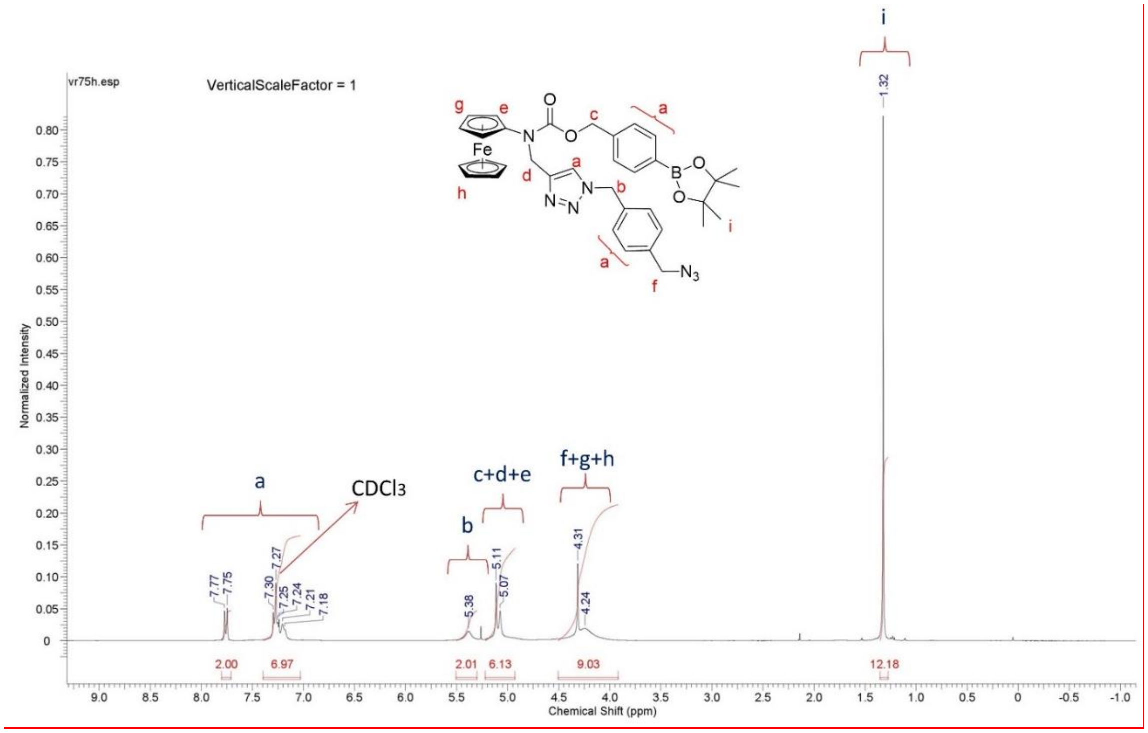

Figure S6. ^{1}H NMR spectrum of intermediate **6** in $CDCl_3$.

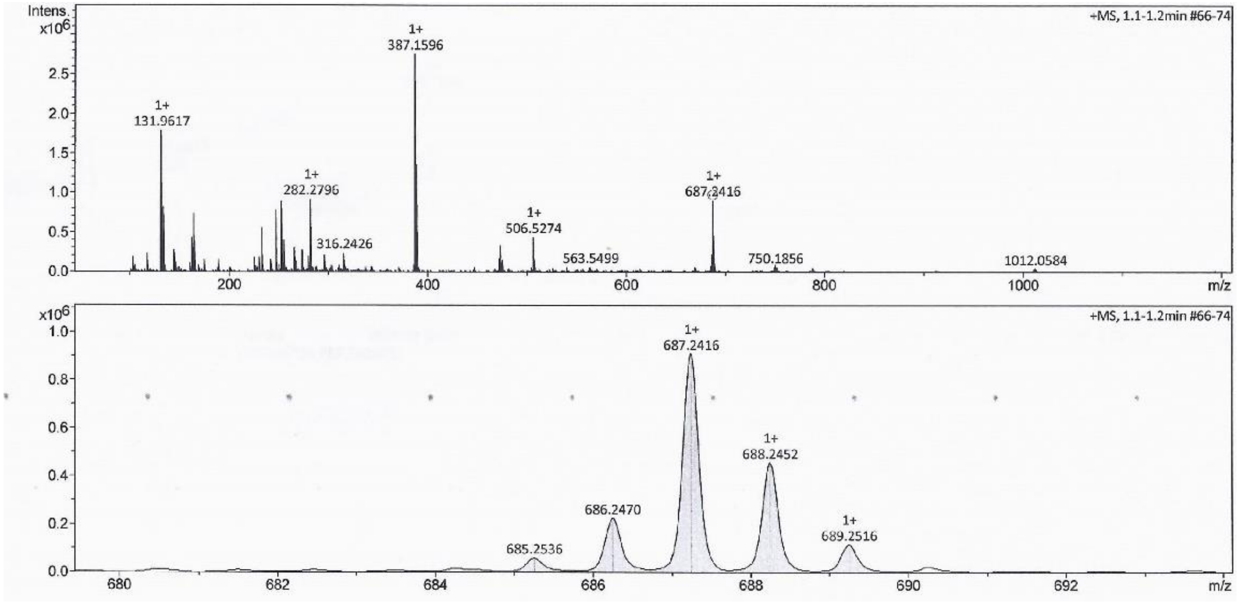

Figure S7. High resolution ESI-TOF mass spectrum of intermediate **6**: upper plot – full experimental spectrum; bottom plot –experimental spectrum zoomed for MP region.

Synthesis of 5-(Trimethylsilyl)ethynyltetramethylrhodamine

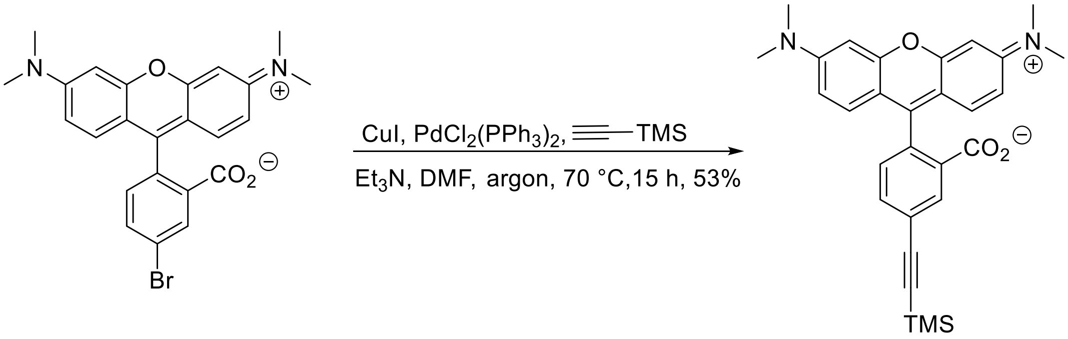

Compound 5-Bromotetramethylrhodamine[6] (760 mg, 1.63 mmol) was dissolved in anhydrous *N,N*-dimethylformamide (15.0 mL) and the solution was degassed with argon for 10 minutes. Afterwards, triethylamine (1.50 mL), copper (I) iodide (31.0 mg, 0.16 mmol), trimethylsilylacetylene (1.16 mL, 8.15 mmol) and bis(triphenylphosphine)palladium (II) dichloride (229 mg, 0.33 mmol) were added under argon atmosphere and the reaction mixture was stirred at 70 °C for 15 hours. The reaction was monitored by LC-MS. Upon completion, the solvent was removed under reduced pressure and the crude product was purified by column chromatography

(dichloromethane / methanol = 10:1 → 7:1) to afford the product 5-(trimethylsilyl)ethynyltetramethylrhodamine (418 mg, 0.87 mmol, 53%) as a purple solid. Thin layer chromatography (TLC), R_f= 0.5 (stationary phase: silica, eluent: CH_2Cl_2/CH_3OH, 7/1, v/v). ^1H NMR (400 MHz, $CDCl_3$): δ (ppm) = 0.28 (s, 9 H), 2.99 (s, 12 H), 6.40 (dd, *J* = 2.6 Hz, *J* = 8.9 Hz, 2 H), 6.48 (d, *J* = 2.5 Hz, 2H), 6.60 (d, *J* = 8.9 Hz, 2 H), 7.10 (dd, *J* = 0.8 Hz, *J* = 7.9 Hz, 1 H), 7.69 (dd, *J* = 1.5 Hz, *J* = 7.9 Hz, 1 H), 8.08 (s, 1 H). DEPTQ (101 MHz, $CDCl_3$): δ (ppm) = 0.0, 40.4, 96.8, 98.6, 103.4, 106.8, 109.0, 124.4, 124.8, 128.3, 128.7, 128.9, 137.8, 152.5, 153.2, 168.9. High resolution ESI-mass spectrometry (positive mode): calcd. for $C_{29}H_{30}N_2O_3Si$ $[M+H]^+$: 483.2098, found: 483.2103.

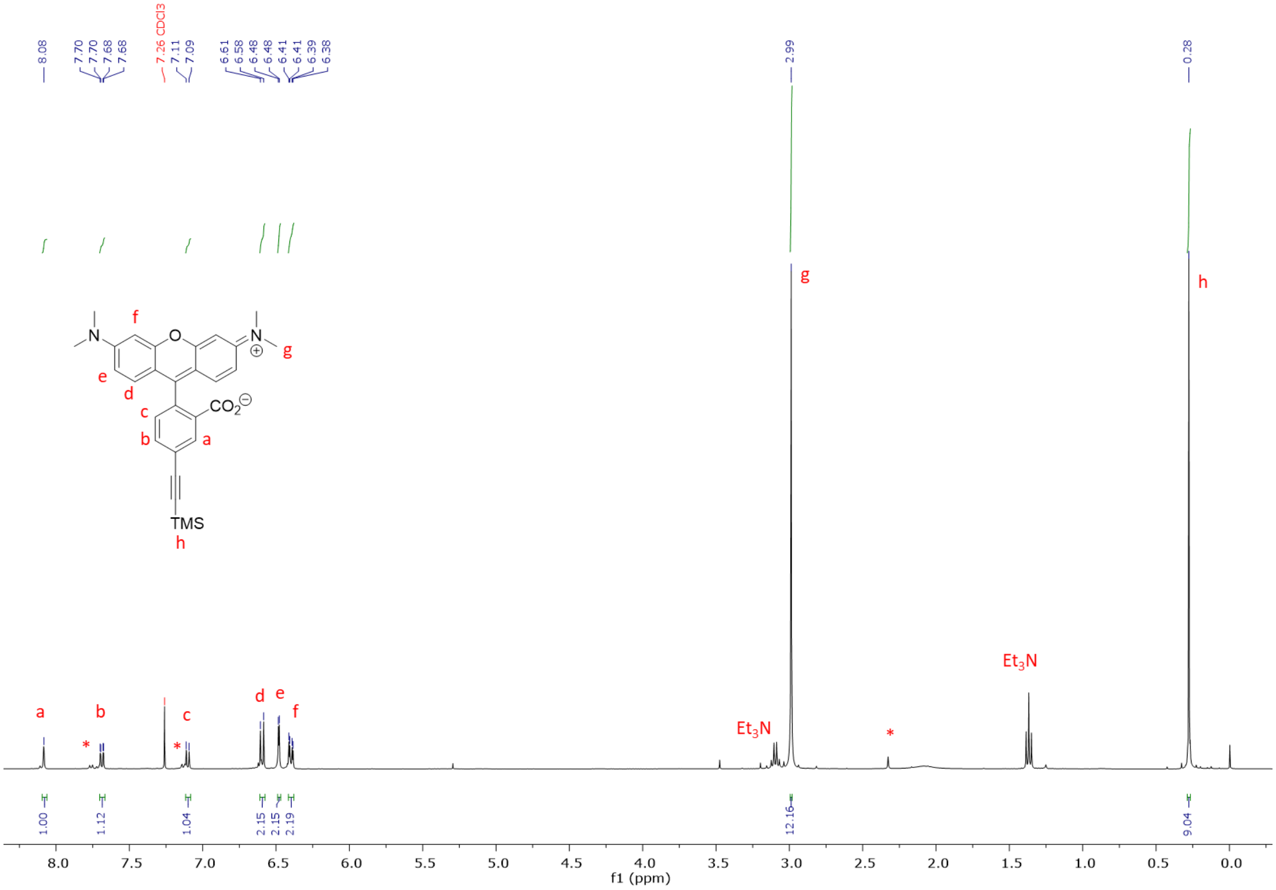

Figure S8: ^1H NMR spectrum of 5-(trimethylsilyl)ethynyltetramethylrhodamine in $CDCl_3$. Impurities are marked with asterisk.

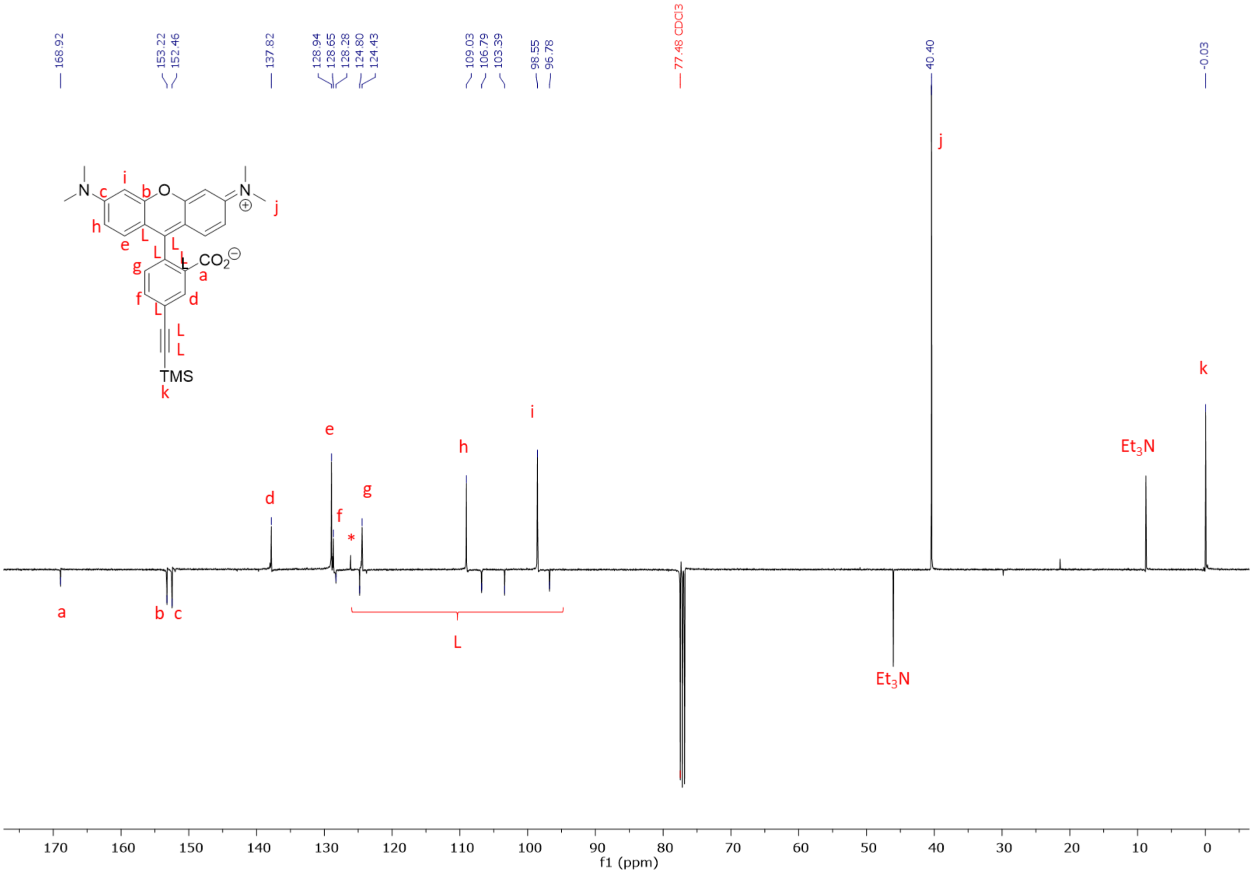

Figure S9: DEPTQ spectrum of 5-(trimethylsilyl)ethynyltetramethylrhodamine in $CDCl_3$. Impurities are marked with asterisk.

Synthesis of intermediate **13**

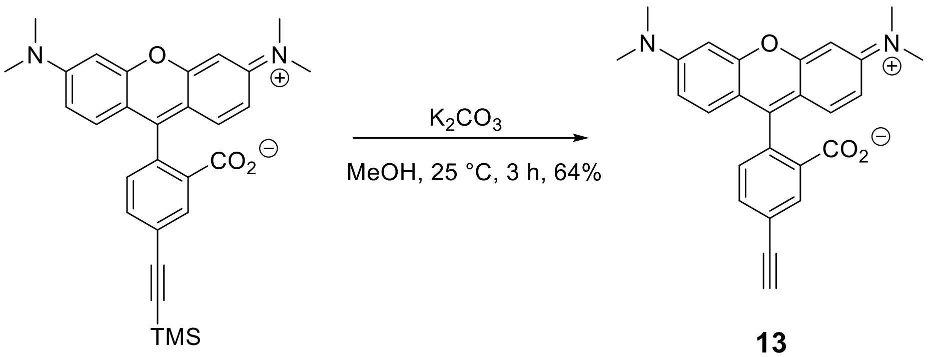

13

Compound 5-(trimethylsilyl)ethynyltetramethylrhodamine (358 mg, 0.74 mmol) was dissolved in methanol (40.0 mL) and potassium carbonate (460 mg, 3.33 mmol) was

added. The reaction was monitored by LC-MS. After stirring at room temperature for 3 hours, the solvent was removed under reduced pressure and the crude product was purified by column chromatography (dichloromethane / methanol = 10:1 → 5:1) to yield intermediate 13 (191 mg, 0.47 mmol, 64 %) as a purple solid. Thin layer chromatography (TLC), R_f= 0.4 (stationary phase: silica, eluent: CH_2Cl_2/CH_3OH, 7/1, v/v). ^{1}H NMR (400 MHz, $CDCl_3$): δ (ppm) = 2.98 (s, 12 H), 3.20 (s, 1 H), 6.40 (dd, *J* = 2.5 Hz, *J* = 8.9 Hz 2 H), 6.48 (d, *J* = 2.6 Hz, 2 H), 6.60 (d, *J* = 8.8 Hz, 2 H), 7.13 (dd, *J* = 0.7, *J* = 7.9 Hz, 1 H), 7.71 (dd, *J* = 1.5, *J* = 7.9 Hz, 1 H), 8.10 (s, 1 H). DEPTQ (101 MHz, $CDCl_3$): δ (ppm) = 40.4, 79.2, 98.6, 106.6, 109.0, 123.8, 124.5, 128.2, 128.8, 128.9, 138.1, 152.4, 153.2, 168.9. ^{13}C-NMR (151 MHz, $CDCl_3$): δ (ppm) = 40.3, 79.1, 82.1, 98.4, 106.7, 109.0, 123.7, 124.5, 128.4, 128.8, 137.8, 152.4, 153.2, 168.7. High resolution ESI-mass spectrometry calcd. for $C_{26}H_{22}N_2O_3$ $[M+H]^+$: 411.1703, found: 411.1701.

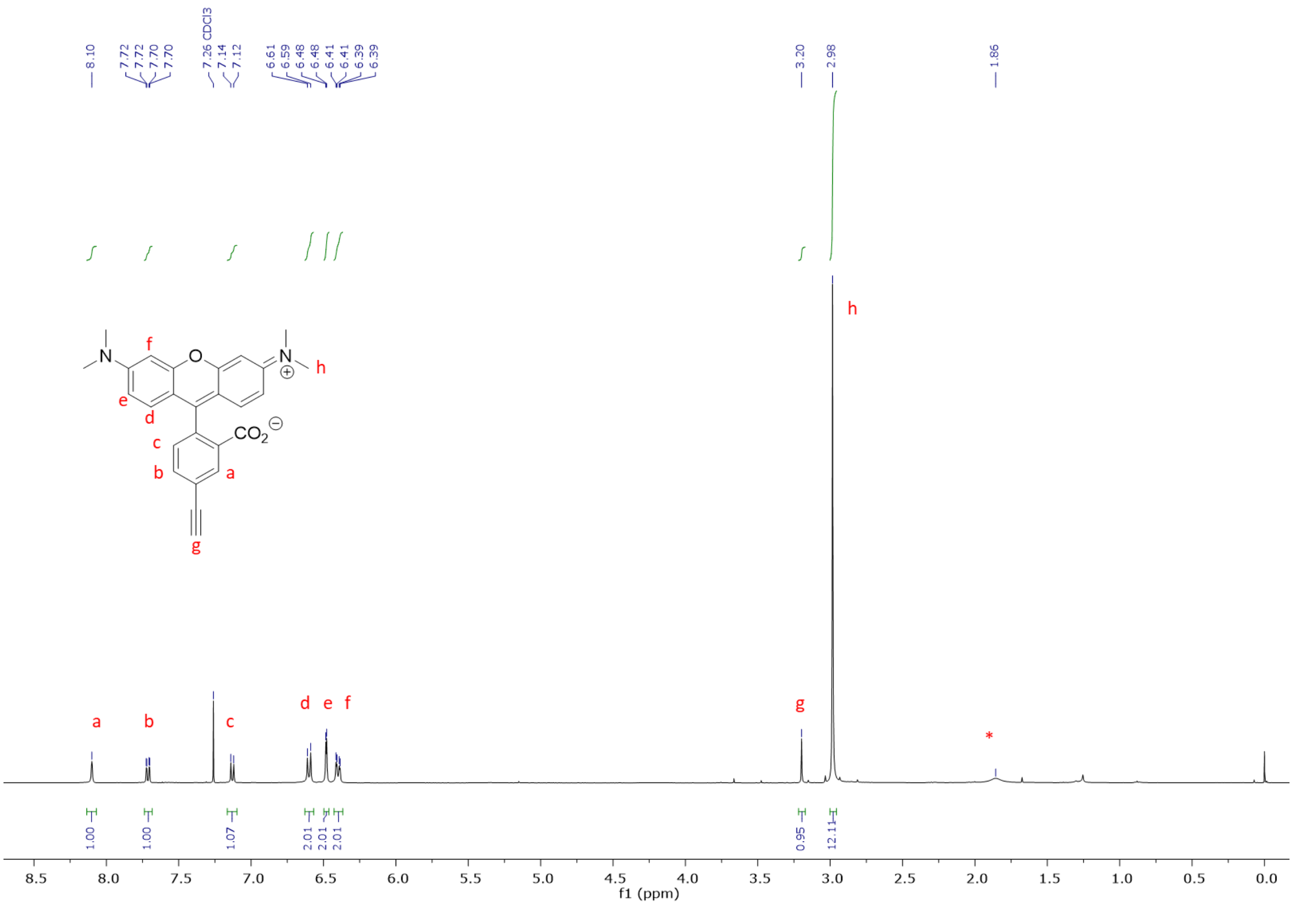

Figure S10: ^{1}H NMR spectrum of intermediate **13** in $CDCl_3$. Impurities are marked with asterisk.

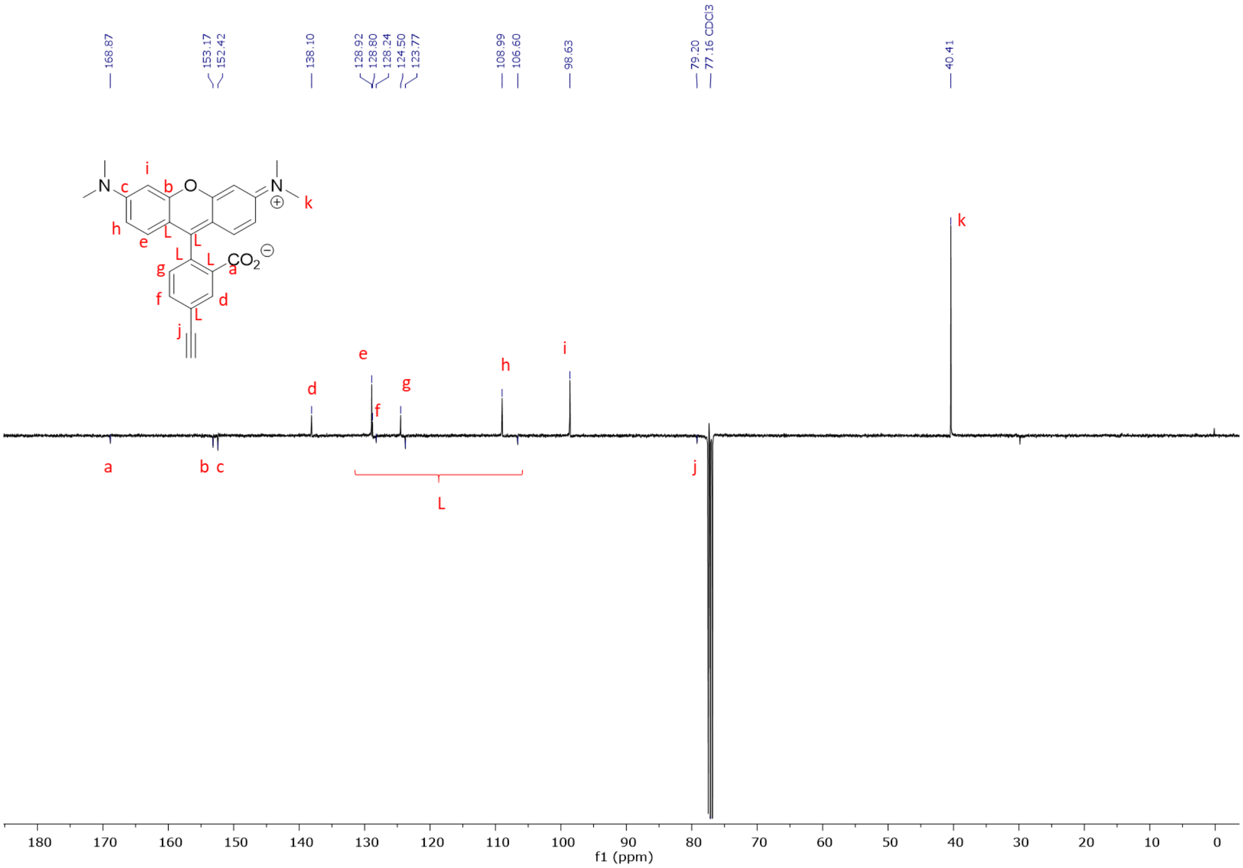

Figure S11: DEPTQ spectrum of intermediate **13** in $CDCl_3$.

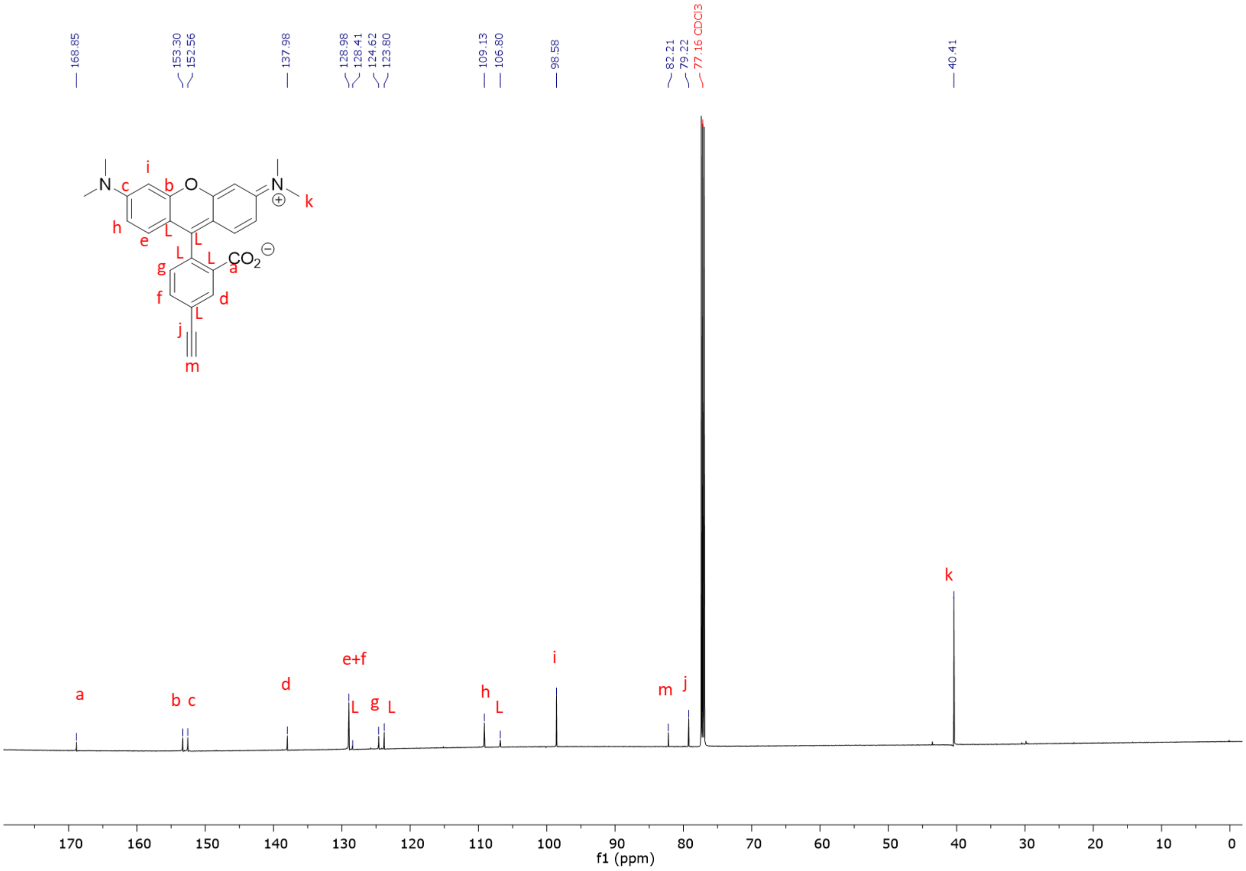

Figure S12: ^{13}C NMR spectrum of intermediate **13** in $CDCl_3$.

Synthesis of prodrug **7**

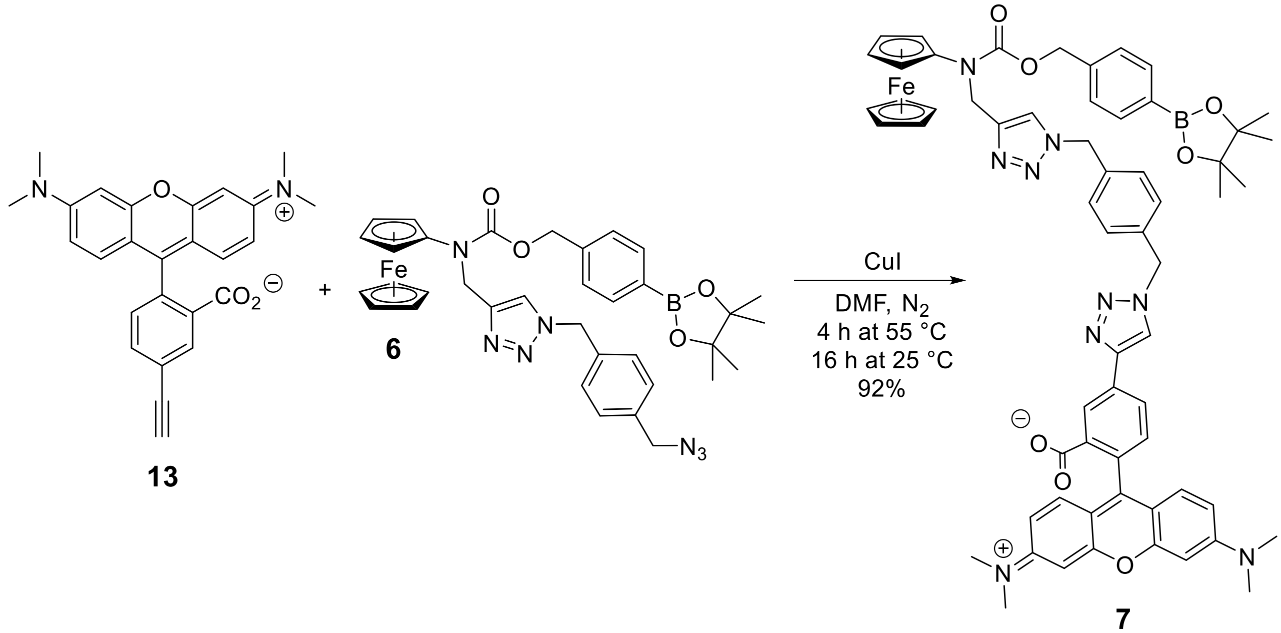

Compound **6** (80 mg, 116 µmol) was dissolved in anhydrous *N*,*N*-dimethylformamide (700 µL) and copper(I)iodide (18mg, 93 µmol) was added. Compound **13** (48 mg, 116 µmol) was added to the reaction mixture by dissolving it in anhydrous *N*,*N*-dimethylformamide (2 mL). The reaction mixture was stirred at 55 °C for 4 h and additional 16 hours at 25 °C. After the reaction completed, solvent was partially removed, and the rest was divided into falcon tubes as 400 µL each and the crude product was precipitated by addition of water followed by centrifugation and decantation (12 mL, 3x). The precipitate was further washed with ethanol (300 µL, 2x) and finally combined by dissolving in CH_2Cl_2. After removal of the solvent, the compound was obtained as dark violet-red solid (92%, 118 mg, 108 µmol). Thin layer chromatography (TLC), R_f= 0.36 (stationary phase: silica, eluent: CH_2Cl_2/CH_3OH, 9/1, v/v). ^{1}H NMR (400 MHz, DMSO-d6) δ 8.84 (s, 1H), 8.24 – 7.95 (m, 3H), 7.77 (d, J = 7.5 Hz, 1H), 7.64 (d, J = 7.4 Hz, 2H), 7.39 – 7.32 (m, 6H), 6.51 (d, J = 20.1 Hz, 6H), 5.67 (s, 2H), 5.58 (s, 2H), 5.16 (s, 2H), 4.91 (s, 2H), 4.51 (s, 2H), 4.04 (s, 5H), 3.98 (s, 2H), 2.96 (s, 12H), 1.27 (s, 12H). High resolution ESI-mass spectrometry (positive mode): calcd. for $C_{61}H_{61}BFeN_9O_7$: 1098.4116; found m/z: 1098.4142. Elemental analysis: calcd (%) for $C_{61}H_{60}BFeN_9O_7{\bullet}4CH_3OH$: C 63.63, H 6.33, N 10.27; found: C 64.02, H 5.94, N 10.61.

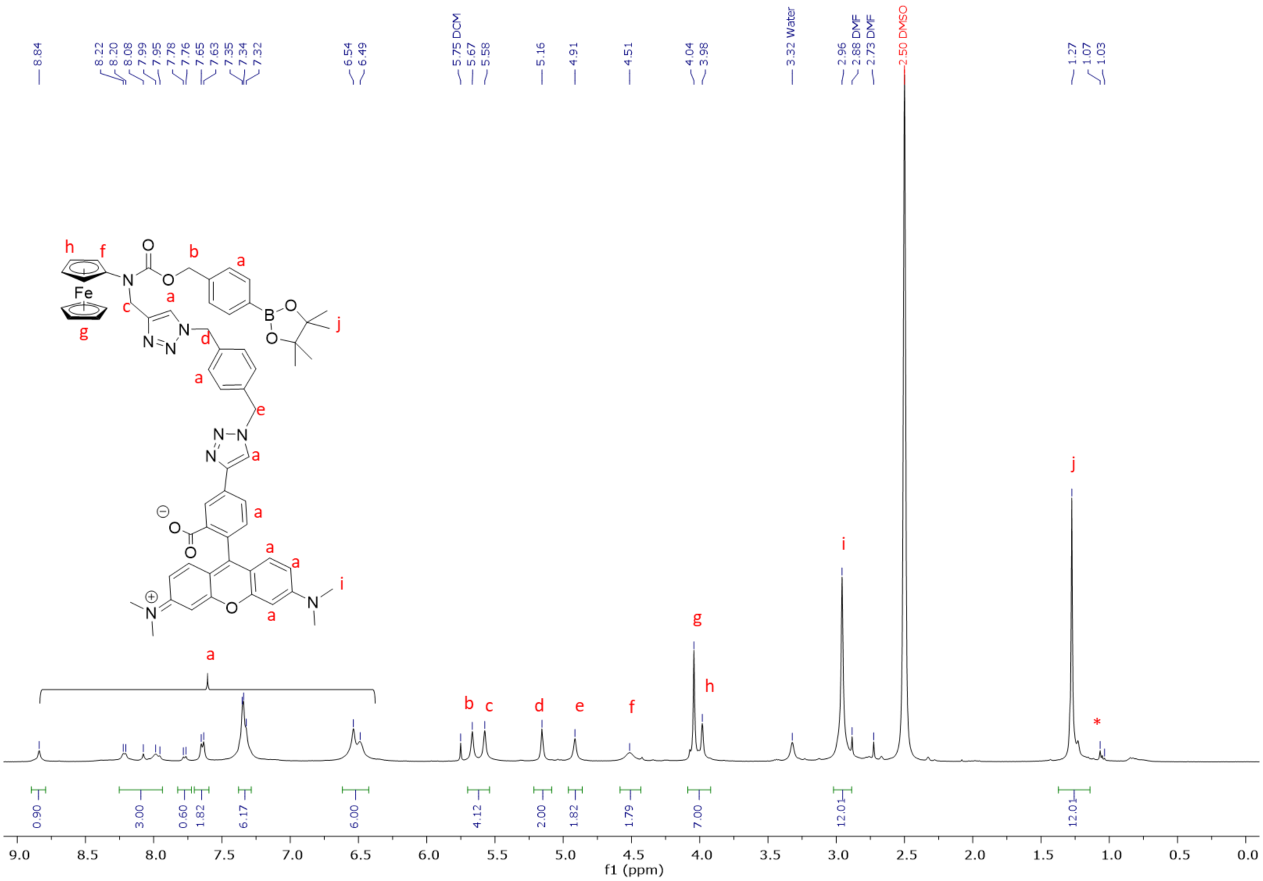

Figure S13. ^{1}H NMR spectrum of prodrug **7** in DMSO-d6. Impurities are marked with asterisk.

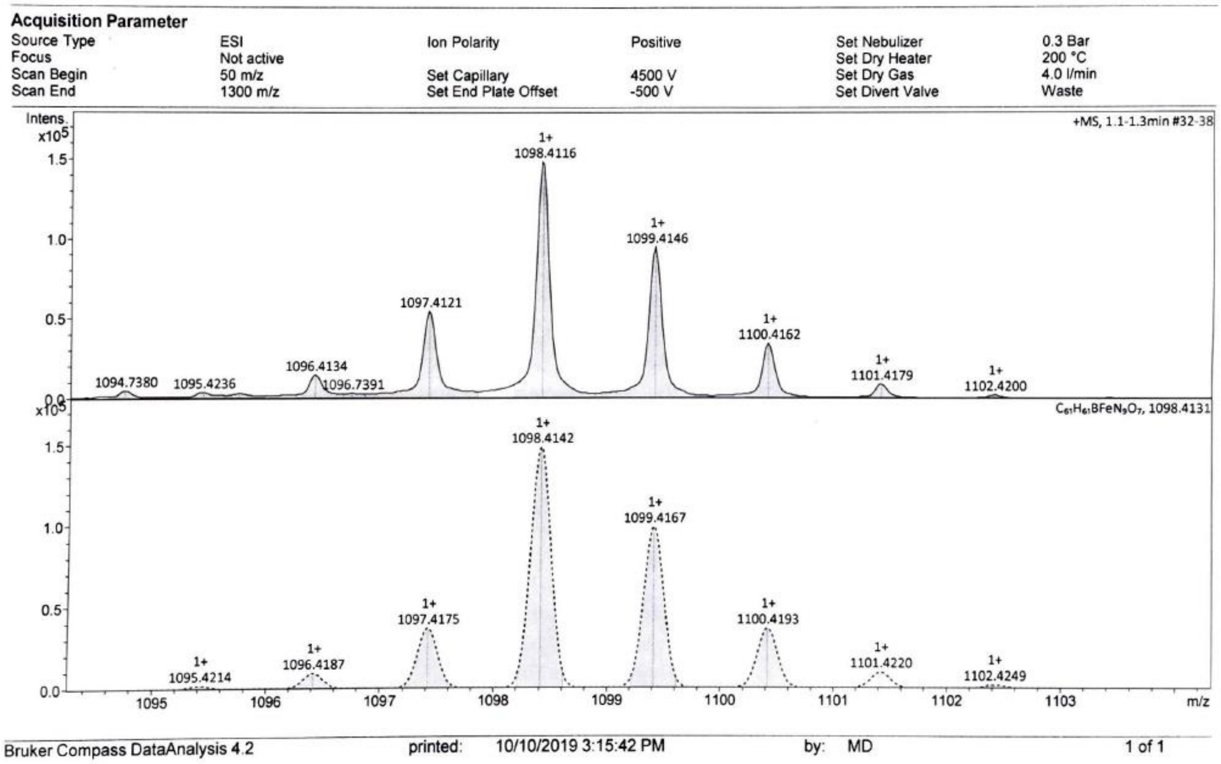

Figure S14. High resolution ESI-TOF mass spectrum of prodrug **7**: upper plot – theoretical spectrum; bottom plot – experimental spectrum.

Additional data not included in the main text of the paper

Table S1. Solubility of prodrugs in aqueous solutions.

Prodrug	Solubility in DPBS, μM	Solubility in FBS, μM
**5**	70 ± 15	130 ± 17
**7**	44 ± 4	47 ± 4
**9**	90 ± 10	114 ± 11

Table S2. Initial rates of DCFH oxidation in the presence of H_2O_2 and prodrugs/controls.

Prodrug/control	$(dF_{525nm}/dt)_0$ (a.u. x min^{-1})[i]
no prodrug added (reference)	0.1 $\pm$ 0.02
Prodrug **5**	7.7 $\pm$ 2.3
Prodrug **8** (known prodrug)[5]	17.1 $\pm$ 4.1
$FeSO_4$ (positive control)	22.3 $\pm$ 5.5

[i] a.u.= arbitrary units.

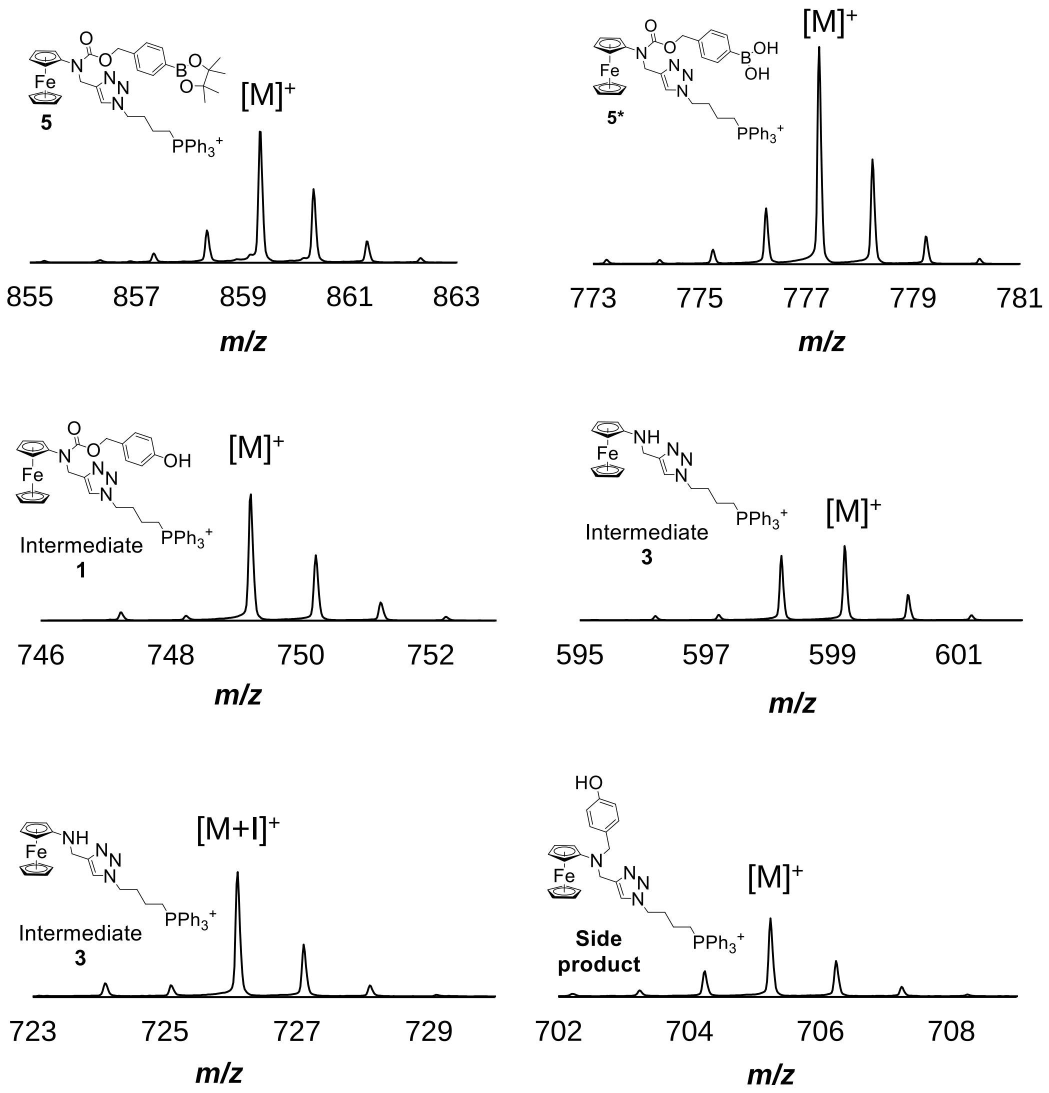

Figure S15. Zoomed in regions of the mass spectrum shown Figure 2B.

Table S3. Uptake of prodrug **5** by BL-2 cells.

Compound	ΔA1	ΔA2	ΔA3	ΔA4	ΔA5	ΔA6	ΔA (mean)	Standard deviation
**8**	1.95	1.85	1.79	1.97	1.93	1.93	1.90	0.07
**5**	2.10	1.60	2.33	2.71	1.50	-	2.05	0.50
**9**	1.01	1.02	0.96	0.96	0.97	0.97	0.98	0.03
Cells only	0.47	0.53	-	0.61	0.55	-	0.54	0.06

References

1. Reshetnikov, V.; Daum, S.; Mokhir, A. *Chem. Eur. J.* **2017**, *23(24)*, 5678-5681.
2. Evoniuk, C. J.; Hill, S. P.; Hansona, K.; Alabugin, I. V. *Chem. Commun.* **2016**, *52*, 7138-7141.
3. Daum, S.; Babiy, S.; Konovalova, H.; Hofer, W.; Shtemenko, A.; Shtemenko, N.; Janko, C.; Alexiou, C.; Mokhir, A. *J. Inorg. Biochem.* **2018**, *178*, 9-17.
4. Chen, X.; Khairallah, G. N.; O'Hair, R. A. J.; Williams, S. J. *Tetrahedron Lett.* **2011**, *52(21)*, 2750-2753.
5. Daum, S.; Reshetnikov, V.; Sisa, M.; Dumych, T.; Lootsik, M. D.; Bilyy, R.; Bila, E.; Janko, C.; Alexiou, C.; Herrmann, M.; Sellner, L.; Mokhir, A. *Angew. Chem. Int. Ed.*, **2017**, *56(49)*, 15545-15549.
6. Deal P. E.; Kulkarni R. U.; Al-Abdullatif S. H.; Miller, E. W. *J. Am. Chem. Soc.* **2016**, *138*, 29, 9085-9088.
